# Trace Elements in Indoor Dust Exposure from Child Development Centers and Health Risk Assessment in Haze and Industrial Areas, Thailand

**DOI:** 10.3390/toxics13070547

**Published:** 2025-06-29

**Authors:** Susira Bootdee, Sopittaporn Sillapapiromsuk, Sawaeng Kawichai

**Affiliations:** 1 Chemical Industrial Process and Environment Program, Faculty of Science, Energy, and Environment, King Mongkut’s University of Technology North Bangkok (Rayong Campus), Rayong 21120, Thailand; susira.b@sciee.kmutnb.ac.th; 2 Department of Environmental Science and Technology, Faculty of Science, Lampang Rajabhat University, Lampang 52100, Thailand; sopittaporn@g.lpru.ac.th; 3 Research Institute for Health Sciences (RIHES), Chiang Mai University, Chiang Mai 50200, Thailand

**Keywords:** indoor school dust, trace elements, health risk assessment, industrial areas, haze pollution

## Abstract

This study aimed to examine trace element concentrations in indoor dust and evaluate health risks in child development centers in haze and industrial areas of Thailand from November 2023 to April 2024. The samples were extracted using a microwave oven and analyzed via ICP-OES. The finding indicated that the levels of As, Cr, Pb, V, Fe, Mn, and Zn in the dust from child development centers in the industrial area were significantly higher than those in the haze area (*p* < 0.05). The presence of trace element contaminants in indoor dust is indicative of anthropogenic sources. Cd and Zn in both areas have shown significantly elevated risks, according to the probable ecological risk factor. Source apportionment identified traffic, road dust, and biomass combustion as the principal sources of pollution in the haze area, while traffic and combustion activities were significant in the industrial area. Non-carcinogenic risk assessments for children exposed to As, Pb, Cu, and Cr revealed potential health risks (HI > 1). Furthermore, the total cancer risk (TCR) linked to As, Cr, and Ni is considered acceptable within the criteria of 10^−6^ to 10^−4^. However, long-term exposure may increase the risk of cancer in children.

## 1. Introduction

The International Agency for Research on Cancer (IARC) confirmed that the inhalation of particulate matter (PM) pollution may cause cancer in humans [1]. Numerous studies have investigated the prevalence of dangerous compounds in indoor dust obtained from residences, educational institutions, industrial locations, and urban environments. These investigations have continually revealed toxic substances in the dust, including VOCs, trace elements, and polycyclic aromatic hydrocarbons (PAHs). Therefore, exposure to dust, especially indoors or outdoors, causes significant negative health effects [2,3,4]. Dust is considered a significant health risk due to its ability to penetrate the respiratory system. It can target specific organs, especially the lungs, potentially resulting in localized damage [5]. Dust particles above 45–63 µm are observed to possess elevated levels of trace elements [6]. Research by Jaafar and Kadhum et al. [7] found that dust particles that are 63 µm in size had high amounts of trace elements: cadmium (Cd) was between 6.5% and 85.8%, nickel (Ni) was from 12.4% to 47.8%, chromium (Cr) ranged from 14.6% to 71.3%, zinc (Zn) was from 18.1% to 66.6%, copper (Cu) was between 22.7% and 81.1%, and lead (Pb) ranged from 0.28% to 61.3%. Moreover, dust particles measuring around 75 µm had a significant association with Pb (10.1% to 76.5%) and Zn (5.8% to 89.5%).

In 2021, the World Health Organization (WHO) reported that exposure to household air pollution was associated with the mortality of more than 500,000 children under five years of age [8]. Since children spend 70–90% of their time indoors, they are relatively more exposed to multiple toxic pollutants in the indoor air, which poses health risks [9]. Several studies have investigated trace elements in indoor dust from nurseries, schools, and universities spanning several countries. The main carcinogenic metal components identified in dust are As, Cd, Cr, Ni, and Pb, with the International Agency for Research on Cancer (IARC) identifying these metals as causes of cancer in humans [1]. Moghtaderi et al. [10] investigated carcinogenic metals in the dust of schools in the megacity of Shiraz, Iran. The concentration of Cr, As, and Pb in dust has significant long-term effects on cancer risks. The presence of carcinogenic metals in the dust at nurseries in Malaysia was identified as Ni, Cr, and Pb, indicating sources released from vehicle emissions, streets, and soil dust [11]. Furthermore, they conducted an investigation into the presence of trace elements in indoor dust at kindergarten schools that are situated near an e-waste recycling area. They reported that Al, V, Mn, Co, Ni, and Zn in the dust at an e-waste recycling area were significantly higher than those in background areas in China [12], while trace elements in kindergarten dust during 2019 and 2020 were found to have Ni, Zn, As, and Ba more than the allowable limits in China [13]. Moreover, an investigation into the toxic metal contamination of dust inside schools in Agra, India, found that Pb, Cr, Cd, and Ni, which are associated with carcinogenic risk, come from industrial activities, automobiles, and fuel combustion [14].

Toxicological studies on trace elements indicate that exposure to Pb, silver (Ag), Cd, and mercury (Hg) in children can increase the risk of developing dyslexia [15]. Igra et al. [16] reported that Cd and Pb exposure for children might impair the risk of reduced growth due to malnutrition. Exposure to Cr and Cd could relate to DNA and chromosome damage, which impairs DNA and interferes with the functionality of DNA repair genes, significantly contributing to its genotoxic effects [7,17,18]. According to the IARC, there is strong evidence connecting chromium (Cr) exposure to lung cancer in humans. The dangerous form, hexavalent chromium (Cr (VI)), can enter both erythrocytes and leukocytes, and there is a strong connection between Cr (VI) exposure and increased death rates from cancers in the nose and nearby sinuses [3]. Moreover, exposure to Pb for children revealed that it is negatively related to hemoglobin and mean corpuscular hemoglobin in red blood cells [19]. The link between exposure to Pb, Hg, Cd, and As and brain development problems in children is a serious concern, leading to conditions like autism spectrum disorder (ASD), attention deficit hyperactivity disorder (ADHD), and learning difficulties [20].

Despite extensive research, there is still insufficient comparative data on the exposure of vulnerable populations to trace element-bound indoor dust related to educational settings across different pollution sources. This research reveals a novel comparative investigation of trace element contamination in dust inside child development centers in Thailand. The research has combined detailed risk assessments and compared the levels of trace elements, their sources, and health risks (including non-cancer, cancer, and effects on the environment) in industrial and biomass-burning areas. This research is critical to establishing environment-specific protective policies and treatments for vulnerable groups, especially children under the age of 5 years.

## 2. Materials and Methods

### 2.1. Sampling Sites

**Figure 1:**
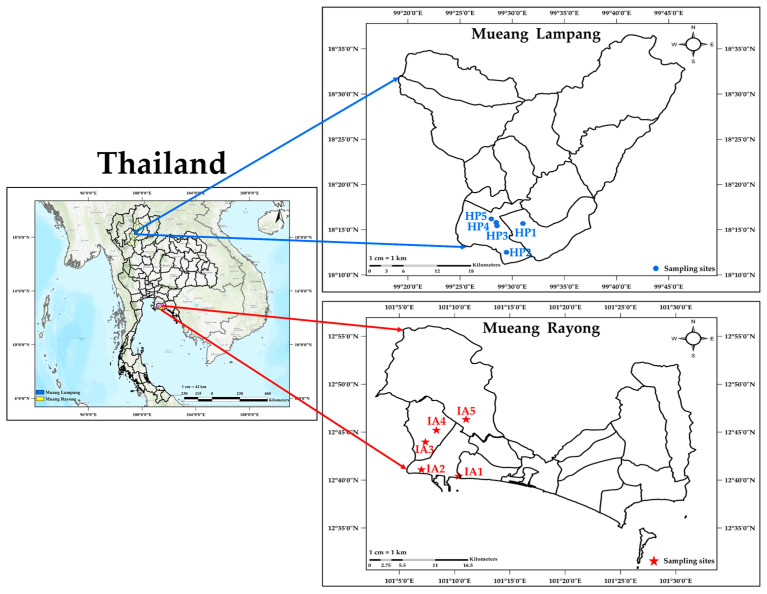
Map of sampling sites in the child development centers in haze and industrial areas, Thailand.

The sampling sites of child development centers were monitored in the haze area in Lampang province (HP), where the main source of pollution is open burning. The Map Ta Phut Town municipality in Rayong province represents the industrial area (IA) in Thailand. The child development center serves as a nursery for children between the ages of 2.5 and 5 years. This research selected and collected the dust samples from five child development centers in each area, as shown in Figure 1. Table 1 displays the details of the sampling process.

HP (HP1-5): Lampang province is essential during the haze period in Thailand. The biomass burning contributed to air pollution in northern Thailand. It is at a latitude of 18°59′53″ north and a longitude of 99°24′26″ east. We selected five child development centers to represent the urban region of Lampang Province. Considerations such as the proximity of child development centers to roadways, residential and commercial buildings, government offices, transportation systems, high-traffic areas, and locations with significant human activity determined the selection of sampling sites.

IA (IA1-5): The sampling site is located at the Map Ta Phut industrial estate, an important industrial complex situated on the eastern coast of the Gulf of Thailand. The industrial estate is located in Rayong Province at a latitude of 12°40′6″ north and a longitude of 101°16′30″ east. It is mainly defined by the presence of petrochemical and chemical manufacturing facilities, petroleum refineries, coal-powered generating plants, residential areas, and transportation infrastructure [21].

From November 2023 to April 2024, dust samples were collected from inside child development centers in Lampang and Rayong provinces, making sure to take them at a height above 50 cm from the floor. The indoor dust samples were collected from a variety of surfaces, such as books and document shelves, tables, curtain structures, TV screens, and the frames of doors and windows where we placed the sampler. We also collected dust samples by brushing them into a sealed polyethylene bag. We dried the dust samples in an oven at 105 °C overnight [22,23]. After that, the dust samples were separated using a <63 µm sieve and kept in desiccators.

### 2.2. Extraction and Analysis of Trace Elements-Bound Dust Sample

After the collection of dust, indoor dust samples at 50 mg were weighed before being dissolved in mixtures of 10 mL of nitric acid (65% HNO_3_) and 2 mL of hydrochloric acid (98% HCl) in a microwave oven (Milestone Ethos Up, Sorisole, Bergamo, Italy). The digestion process involved raising the temperature to 210 °C at 1800 W power and holding it there for 35 min (Methods library for SK-15 ET high-pressure rotor). Finally, the solution was filtered through a nylon syringe filter (0.45 µm and 13 mm) and diluted with 25 mL of deionized water. Dust samples were extracted and analyzed from three repetitions for the sampling site. Additionally, an inductively coupled plasma-optical spectrometer (ICP-OES, Agilent 5800, Santa Clara, CA, USA) was used to analyze ten elements after digestion preparation, including arsenic (As), cadmium (Cd), chromium (Cr), nickel (Ni), lead (Pb), vanadium (V), copper (Cu), iron (Fe), manganese (Mn), and zinc (Zn).

The accuracy of the analysis and extraction conditions was verified using 50 mg of the National Institute of Standard and Technology (NIST, USA) Standard Reference Material 1648a (SRM 1648a) and Standard Reference Material 2583 (SRM 2583) for trace elements in indoor dust. Table S1 shows the element recoveries from five replications with SRM methods. SRM element recoveries ranged from 69.9% (Cr) to 94.9% (Cu). The limit of detection (LOD) for elements ranged from 0.051 to 0.200 mg kg^−1^, and the limit of quantitation (LOQ) was 0.172–0.667 mg kg^−1^, as shown in Table S2.

### 2.3. Pollution Assessment of Trace Elements in Dust

#### 2.3.1. Enrichment Factor

The enrichment factor (EF) is a tool used in environmental studies to evaluate the relation of specific metals that are concentrated in dust relative to their natural abundance. The concentration of pollution is caused by indoor dust-bound trace elements that originate from natural or anthropogenic processes [24,25] and is defined in Equation (1).

(1) $$\mathrm{EF}=\frac{{\left(\frac{C_{i}}{C_{Fe}}\right)}_{Sample}}{{\left(\frac{C_{i}}{C_{Fe}}\right)}_{Crust}}$$

where *C_i_* represents the average concentration of each specific metal in indoor dust (the sample) and the Earth’s crust, whereas *C_Fe_* denotes the mean concentration of Fe in both the indoor dust and the surface of the Earth. Several researchers have effectively utilized Fe as a widely accepted reference element for geochemical normalization [24,25,26]. The concentration of trace elements and elements in the crust was determined using the topsoil samples at Rayong province, incorporating data from both general soil and paddy soils in Thailand [27,28], as illustrated in Table S3. Researchers can more accurately assess the influence of anthropogenic activities on trace elements concentrations in diverse ecosystems by examining five categories of EF in Table S4.

#### 2.3.2. Geo-Accumulation Index

The geo-accumulation index (*I_geo_*) is widely used to assess anthropogenic impacts on the environment. It has been used to evaluate the level of trace elements pollution in dust or particulate matter in the atmosphere by comparing current concentrations with pre-industrial baseline levels [25,29,30]. The *I_geo_* index is identified using Equation (2).

(2) $$I_{geo}=\log_{2}\frac{C_{n}}{\left(1.5\times B_{n}\right)}$$

In this case, *C_n_* is the indoor dust trace elements content (mg kg^−1^) and *B_n_* is the background element content (mg kg^−1^). Background values from topsoil in Rayong province and general soil and paddy soils in Thailand have been reported in the past [27,28,31] (Table S3). Table S4 delineates the classification of *I_geo_* levels into seven groups [29].

#### 2.3.3. Assessment of the Potential Ecological Risk Index (RI)

The potential ecological risk index (RI) assesses the cumulative potential ecological risk associated with trace elements in dust samples or environmental media (such as soil, sediment, dust, or water). The RI is a summative index that evaluates the cumulative potential ecological risk posed by multiple contaminants in a given environment, taking into account their contamination levels, toxic effects, and ecological impact [32,33,34,35]. The RI can be calculated by Equation (3).

(3) $$\mathrm{RI}=\sum_{i=1}^{n}E_{r}^{i}$$

(4) $$E_{r}^{i}=T_{r}^{i}\times \;\left(\frac{C_{n}}{B_{n}}\right)$$

where $E_{r}^{i}$ represents the potential ecological risk factor of trace element (i) in dust, while $T_{r}^{i}$ denotes its toxicity coefficient. This factor is calculated by multiplying $T_{r}^{i}$ with *C_i_* and *B_n_*, where *C_n_* is the concentration of individual trace element in dust and *B_n_* represents the background value of soil, consistent with its meaning in the previous equations (Equation (4)). The toxicity coefficients ($T_{r}^{i}$) for trace elements were As = 10; Cd = 30, Cr = V = 2, Pb = Cu = Ni = 5, and Fe = Mn = Zn = 1 [32,34,36]. Table S5 displays the classification levels and the relationship between Er, RI, and degree.

### 2.4. Health Risk Assessment

#### 2.4.1. Non-Carcinogenic Risk Assessment

The non-carcinogenic risk assessment includes determining the possibility of adverse health effects resulting from exposure to hazardous substances that are not associated with cancer. A calculation known as the hazard quotient (HQ) provides a rough estimate of the level of risk, unrelated to cancer, associated with inhaling pollutants [4]. The calculation involves dividing the average daily dose (ADD) by the reference dosage (RfD) to determine the daily metal inhalation (Table S6), as shown in Equation (5).

(5) $$\mathrm{HQ}=\frac{\mathrm{ADD}}{\mathrm{RfD}}$$

(6) $${\mathrm{ADD}}_{Inhalation}=C_{Metal}\;\times \;\left[\frac{\;\mathrm{InhR}\;\times \;\mathrm{EF}\;\times \;\mathrm{ED}}{\;\mathrm{PEF}\;\times \;\mathrm{BW}\;\times \;\mathrm{AT}}\right]$$

(7) $${\mathrm{ADD}}_{\mathrm{Ingestion}}=C_{\mathrm{Metal}}\;\times \;\left[\frac{\;\mathrm{IngR}\;\times \;\mathrm{EF}\;\times \;\mathrm{ED}}{\;\mathrm{BW}\;\times \;\mathrm{AT}}\right]\times \mathrm{CF}$$

(8) $${\mathrm{ADD}}_{\mathrm{Dermal}}=C_{\mathrm{Metal}}\;\times \;\left[\frac{\;\mathrm{SA}\;\times \;\mathrm{SL}\;\times \;\mathrm{ABS}\;\times \;\mathrm{EF}\;\times \;\mathrm{ED}}{\;\mathrm{BW}\;\times \;\mathrm{AT}}\right]\times \mathrm{CF}$$

(9) $$\mathrm{HI}={HQ}_{\mathrm{Ingestion}}+{HQ}_{\mathrm{Inhalation}}+{HQ}_{\mathrm{Dermal}}$$

where ADD is the average daily dose (mg kg^−1^ day^−1^) and RfD is the reference dosage (mg kg^−1^ day^−1^). The term *C_Metal_* refers to the concentration of carcinogenic metals in dust (mg kg^−1^). Equations (6)–(8) describe how to calculate ADD for exposure to trace elements in dust. Table S7 presents comprehensive details regarding the parameter values used for assessing ADD in the health risk assessment [37,38]. The Hazard Index (HI) is the sum of the Hazard Quotients (HQs) for the exposure routes associated with trace elements in indoor dust, as shown in Equation (9). If HQ and HI are greater than 1.0, there is a potential for non-carcinogenic effects to occur. Conversely, an HQ and HI of less than 1.0 indicates no hazards or only negligible risks.

#### 2.4.2. Carcinogenic Risk Assessment

Carcinogenic risk assessment is the process of evaluating the potential for a chemical or pollutant to cause cancer in humans. Arsenic (As), Cd, Cr, Ni, and Pb are classified as toxic metals by the International Agency for Research on Cancer [3]. This assessment is critical for determining the likelihood and magnitude of cancer risk (CR) associated with exposure to a variety of substances. The CR is calculated by multiplying the average daily dose (ADD) for carcinogenic metal exposure by the cancer slope factor (SF, per mg kg^−1^ day^−1^), as shown in Equation (10) [38,39]. Moreover, total cancer risk (TCR) is the sum of the risks associated with all exposure pathways (Equation (11)). The parameters are presented in Tables S6 and S7.

(10) CR=ADD × SF

(11) $$\mathrm{TCR}={CR}_{\mathrm{Inhalation}}+{CR}_{\mathrm{Ingestion}}+{CR}_{\mathrm{Dermal}}$$

According to TCR and CR risk categories, values less than 10^−6^ are considered insignificant, whereas values greater than 10^−4^ indicate an unacceptable risk. A risk that is considered acceptable or manageable is indicated by TCR and CR values ranging from 10^−6^ to 10^−4^.

### 2.5. Data Analysis

Statistical analysis using the Kolmogorov–Smirnov test in SPSS (IBM Corp., IBM SPSS Statistics for Windows, Version 29.0, Armonk, New York, NY, USA) indicated a non-normal distribution of trace elements in indoor dust in both the haze and industrial areas of Thailand. The Mann-Whitney U test was used to compare individual trace element concentrations in indoor dust at child development centers between the haze and the industrial city. Additionally, the Kruskal–Wallis test was conducted to assess the mean differences in trace element levels in indoor dust among child development centers in each area. Principal Component Analysis (PCA) with rotated factor loading was used for the source apportionment of trace elements in dust inside child development centers in both the haze and industrial areas of Thailand.

## 3. Results and Discussion

### 3.1. Concentration of Trace Elements in Indoor Dust

Table 2 presents the median and average concentrations of trace elements found in dust samples collected from child development centers in a haze- and industry-affected city in Thailand. The concentrations of As, Cd, Cr, Pb, Ni, V, Cu, Fe, Mn, and Zn in indoor dust in child development centers in the haze area were 7.4 ± 0.9 to 14.3 ± 1.0 mg kg^−1^, 1.0 ± 0.01 to 2.0 ± 0.1 mg kg^−1^, 30.1 ± 1.3 to 123.4 ± 14.8 mg kg^−1^, 35.4 ± 0.5 to 351.1 ± 18.3 mg kg^−1^, 22.4 ± 1.5 to 83.1 ± 2.9 mg kg^−1^, 17.3 ± 0.6 to 27.6 ± 0.5 mg kg^−1^, 37.8 ± 3.5 to 5875 ± 384.2 mg kg^−1^, 9316 ± 183.5 to 17,270 ± 256.6 mg kg^−1^, 343.7 ± 10.9 to 637.5 ± 6.1 mg kg^−1^, and 196.4 ± 6.3 to 3203 ± 181.7 mg kg^−1^, respectively.

In the industrial area, the indoor dust levels in child development centers in descending order were Fe (16,438 ± 455.9 to 23,619 ± 315.5 mg kg^−1^) > Zn (410.7 ± 10.5 to 5114 ± 60.3 mg kg^−1^) > Mn (428.5 ± 8.8 to 754.4 ± 13.3 mg kg^−1^) > Cr (58.0 ± 6.7 to 281.4 ± 3.5 mg kg^−1^) > Ni (37.7 ± 4.9 to 253.5 ± 0.6 mg kg^−1^) > Pb (57.7 ± 1.5 to 112.0 ± 11.5 mg kg^−1^) > Cu (79.9 ± 1.4 to 99.2 ± 8.9 mg kg^−1^) > V (26.9 ± 0.9 to 47.8 ± 1.3 mg kg^−1^) > As (14.4 ± 0.8 to 49.2 ± 2.4 mg kg^−1^) > Cd (0.6 ± 0.1 to 3.2 ± 0.1 mg kg^−1^). The highest levels of Pb, Cu, Fe, and Zn in indoor dust were found in the HP3 and IA5 samples, which were taken in heavy traffic and soil dust emissions. Both sampling sites are near a highway. Moreover, the HP3 is near a construction and landfilling supply store, which supplies soil for development to land leveling. In a previous study, they reported that Pb, Cu, Fe, and Zn were released from traffic emissions and road dust [12,40,41]. Moreover, soil and the earth’s crust may emit Cu and Zn in dust [42]. However, there was no significant difference in the levels of trace elements in indoor dust at child development centers between the haze and the industrial area (*p* > 0.05). The trace elements in dust from indoor child development centers in both the haze and industrial areas at each site were not significantly different (*p* > 0.05). Table 2 shows that the amounts of As, Cr, Pb, V, Fe, Mn, and Zn in the dust inside child development centers in the industrial area were significantly higher than those in the haze area (*p* < 0.05).

Table 3 displays the concentrations of trace elements in indoor dust from schools and kindergartens, based on several studies. The previous study examined the buildup of trace element-contaminated dust in an indoor school located in the industrial area of Puchuncaví and Quintero City, Chile [43]. The research showed that the levels of As, Cr, V, and Cu in the dust were 1–2, 3–5, 8–10, and 2–54 times higher than what was found in child development centers in Thailand’s industrial area. However, we found similar levels of Cd, Pb, Ni, Mn, and Zn in the dust compared to this study. The carcinogenic metals, including As, Cd, Cr, Pb, and Ni, were identified as contaminants in indoor kindergarten dust in areas involved in e-waste recycling and industrial cities. Zhang et al. [13] and Parra et al. [43] ranked the carcinogenic metals in the following order: Cr > Ni > Pb > As > Cd. A study conducted in the Islamic Republic of Iran analyzed trace elements in classroom dust samples. The study discovered that indoor environments had the highest levels of Fe, Mn, Pb, Zn, Cr, Cu, Ni, Co, As, and Cd. Iron (Fe) was the metal that came from the Earth’s crust and vehicle emissions, which were similar to this study. Moreover, the carcinogenic metals in classroom dust samples in the following order were Pb (258.8 ± 268.2 mg kg^−1^) > Cr (172.8 ± 122.1 mg kg^−1^) > Ni (50.1 ± 22.5 mg kg^−1^) > As (2.8 ± 1.7 mg kg^−1^), and Cd (1.0 ± 2.3 mg kg^−1^). Notably, the indoor levels of lead (Pb) were higher than other carcinogenic metals, likely due to the schools being located near major streets with heavy traffic [10]. Wang et al. [44] reported the concentrations of trace elements in <63 µm dust samples from an indoor kindergarten near Beijing, China. Researchers found that the levels of Cd (0.4 ± 0.2 mg kg^−1^), Pb (38.1 ± 18.8 mg kg^−1^), Ni (25.6 ± 6.5 mg kg^−1^), Cu (41.2 ± 16.9 mg kg^−1^), and Zn (239.0 ± 112.4 mg kg^−1^) were lower than in this study. The main sources of these trace elements were traffic emissions and mixtures with natural soil. Additionally, we discovered that the As levels in indoor dust at child development centers in both the haze and the industrial area were higher than the Pollution Control Department (PCD) standard of 6 mg kg^−1^, although the values did not surpass the 27 mg kg^−1^ level found in the soil of an industrial estate in Thailand [45,46].

### 3.2. Assessment of Trace Elements Pollution in Indoor Dust

**Figure 2:**
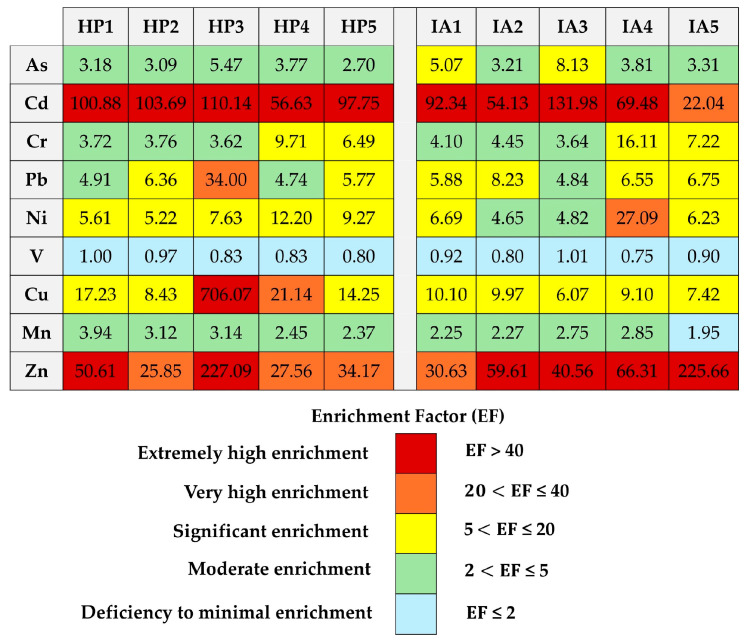
Enrichment factor (EF) levels of the haze (HP) and industrial area (IA) in Thailand.

**Figure 3:**
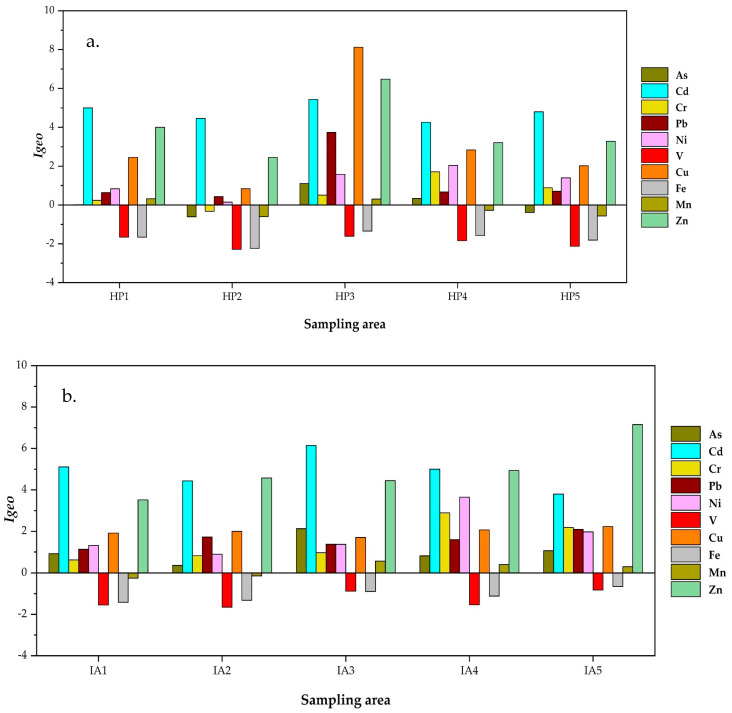
Levels of the geo-accumulation index (*I_geo_*) in both areas: (**a**) haze (HP) and (**b**) industrial area (IA) in Thailand.

Assessing trace element pollution associated with human activities can be beneficial for managing risks in child development centers during the haze and in industrial areas. However, they may be less effective in addressing naturally occurring trace elements. Figure 2 shows how enrichment factor (EF) values can be used to determine how many trace elements and elements are present in industrial areas that are affected by human activity. Figure 3 shows the *I_geo_* levels.

Trace elements were found in child development centers in Thailand. EF levels greater than 2 indicate that the metals originated from human activities, while levels less than or equal to 2 suggest they came from natural sources, according to studies by Zhang et al. [13] and Somsunun et al. [48]. Figure 2 illustrates that the enrichment factor levels of trace elements, including As, Cd, Cr, Pb, Ni, Cu, Mn, and Zn, in indoor dust, surpassed acceptable limits at child development centers in both investigated locations, suggesting anthropogenic influence. The highest EF levels exceeding 20 for trace elements in indoor dust at child development centers in both areas were observed for Cd and Zn. The EF levels followed the same pattern as the *Igeo* levels. *I_geo_* > 3 levels were found for Cd and Zn in indoor dust, which means there is a lot of pollution at child development centers in both areas (Figure 3a,b). The high levels of Cd in indoor dust may come from industrial sources like stabilizers used to make plastic, corrosion-resistant coatings, Ni-Cd batteries, glassware coloring, building materials, and tires [43,48] Additionally, Zn has been linked to emissions from motor vehicles, as well as the steel and galvanization industry, which prevents metal corrosion in indoor dust from schools and homes [48,49,50,51]. Additionally, the highest EF (>20) and *I_geo_* (>5) levels of Cu in indoor dust were observed at the HP3 site. Previous studies investigated the source of Cu in dust inside schools, homes, and residents and found that it was road dust, motor vehicles, the alloy industry (such as bronze and brass), wires, plating, fabric preservation, and wood preservation [11,48,50,52]. Therefore, the presence of trace elements in indoor dust at child development centers in both the haze-affected and industrial areas of Thailand is likely attributed to human activities rather than natural sources.

Our assessment of the potential ecological risk index (RI) and the potential ecological risk factor $\left(E_{r}^{i}\right)$ risks in child development centers in the haze and the industrial areas in Thailand is shown in Table 4. The results indicated that cadmium (Cd) concentrations in indoor dust from child development centers in both areas posed a very high risk (≥320). In contrast, most other trace elements in dust were categorized as low risk (<40). Additionally, copper (Cu) levels in indoor dust were also identified as posing a very high risk (≥320), while zinc (Zn) levels observed at site HP3 indicated a considerable risk (80 ≤ Zn < 160). In site IA5, zinc concentrations were classified as high risk (160 ≤ Zn < 320). However, the RI assessment used to calculate trace element levels in dust at child development centers in both areas revealed RI values exceeding 600, indicating a high risk. The highest RI values in indoor child development centers were recorded in HP3 for the haze area and IA3 for the industrial area. The reason for this study is that we found that the industrial area (IA3) is located at the primary school of the municipality because of its proximity to the Eastern Economic Corridor (EEC) project. The industrial area (IA3) is near the Eastern Economic Corridor (EEC) project, which surrounds it with construction and land leveling. Meanwhile, the sampling site (HP3) is close to a construction and landfill supply store. It is approximately 153 m away from the highway. Thus, the potential ecological risk of trace elements in indoor dust at child development centers in the haze and the industrial areas in Thailand might originate from transportation and industrial emissions [14,33].

### 3.3. Source Apportionment of Indoor Child Development Centers

The principal component analysis (PCA) was performed to determine the potential sources of trace element contamination in dust samples collected from child development centers in the haze (HP) and the industrial areas (IA), which identified eigenvalues of more than 1. Table 5 presents the rotated component matrix, where factor loadings greater than 0.700 are highlighted to indicate significant contributions.

In the HP dataset, Component Factor 1 (PC1) accounted for 65.7% of the total variance and was strongly associated with As, Cd, Pb, V, Cu, Fe, Mn, and Zn. These elements are commonly linked to mixed sources of traffic emission, road dust, and biomass burning as the dominant contributors in HP sites. Burning biomass fuel released high levels of Pb, Cu, Fe, Zn, and Al [53]. The combustion of biomass pellets and raw biomass fuels emitted As, Pb, and Cu [54]. Previous researchers suggested that the investigation of original school dust in Shiraz, Iran, revealed an abundance of As, Cd, and Pb attributed to industrial operations and combustion of fuel [10]. Moreover, the result of this factor is consistent with the EF values, indicating anthropogenic activities. Therefore, the origin of the haze area could be attributed to a combination of sources such as traffic emissions, road dust, and biomass burning. The second factor (PC2), explaining 22.8% of the variance, was characterized by Cr and Ni, metals often related to industrial emissions or metal processing activities. Cr, Ni, and Zn deposits are from stainless steel utensils, metal objects, paints, and building materials [53]. Han et al. [55] reported that road dust in Auyang, China, including As, Cr, Ni, and Co, was obtained from soil parent material or geogenic origins. The EF values of Cr and Ni in indoor dust at child development centers in the haze area suggested human activities. Therefore, the source of this factor was industrial activities, because Lampang is regarded as one of the top cities in Thailand for ceramic production, providing employment for residents and contributing to the community’s income. The black pigment in ceramic tiles included Cr and Ni [56], and trace elements consisted of ceramic products such as Cd, Cr, Pb, Ni, Cu, Mn, Zn, and Co [57]. According to Tu et al. [58], the ceramic industry in China emits Cr, Ni, and Fe, which are trace elements found in PM2.5.

In the IA dataset, PC1 (Factor 1) explained 39.8% of the variation and had high levels of Pb, Cu, Fe, and Zn, which suggests a strong link with emissions from traffic [52,59]. Non-exhaust sources of dust contamination from electronic recycling sites contributed to the resuspension of road dust, releasing Cu, Fe, and Mn. Furthermore, vehicle-related wear, such as brake, bearing, and tire abrasion, emitted Pb, Cu, Fe, and Zn [59]. On the other hand, the presence of Cd, Pb, Cu, and Zn in indoor dust may be linked to the degradation of building materials, deteriorating paint, and the age of the building [14,40,60]. However, we suggested addressing traffic emissions and road dust, as the majority of child development centers in industrial areas are situated near roads and occasionally open their doors and windows. The second factor (PC2) presented As, Cd, V, and Mn and explained 34.9% of the variance, showing strong associations, which imply a contribution from industrial emissions and fuel combustion-related activities. On the other hand, the EF values of V were less than 2, implying a natural source. However, the primary human-caused source of V emissions in China, accounting for 85%, is the burning of fossil fuels. Vanadium is a valuable indicator of petroleum combustion [61]. Moreover, sources of V and Mn included the iron and steel industry [52,62]. Moghtaderi et al. [10] reported that in the original school dust of Shiraz, Iran, As, Pb, and Cd were found to be released from industrial activities and fuel combustion. The third factor (PC3), which accounted for 19.4% of the variance, was mainly influenced by Cr and Ni, both of which are strongly linked to coal power plants or soil dust. Rout et al. [42] found that dust in the ambient air of Jharia, a coal mining town in India, contained Co, Cr, and Ni, originating from coal dust. Additionally, Liu et al. [63] reported that trace elements present in ambient PM2.5 in rural areas, where raw coal was burned and emitted through chimneys, included As, Cr, Ni, Cu, and Zn. Researchers have investigated the airborne particles in Ulsan, Korea, and found Cd, Cr, Ni, Fe, and Zn as signs of soil dust emissions. In Auyang, China, road dust had As, Cr, Ni, and Co, all of which came from soil or geological sources [52,64]. Consequently, researchers have linked this component to either coal power plants or soil dust sources. Industrial areas (IAs) have been developed for land equalization, building construction, and road construction to facilitate logistics, along with coal-fired power plants to support industrial operations.

### 3.4. Health Risk Assessment of Indoor Dust

#### 3.4.1. Non-Carcinogenic Risk of Indoor Dust

**Figure 4:**
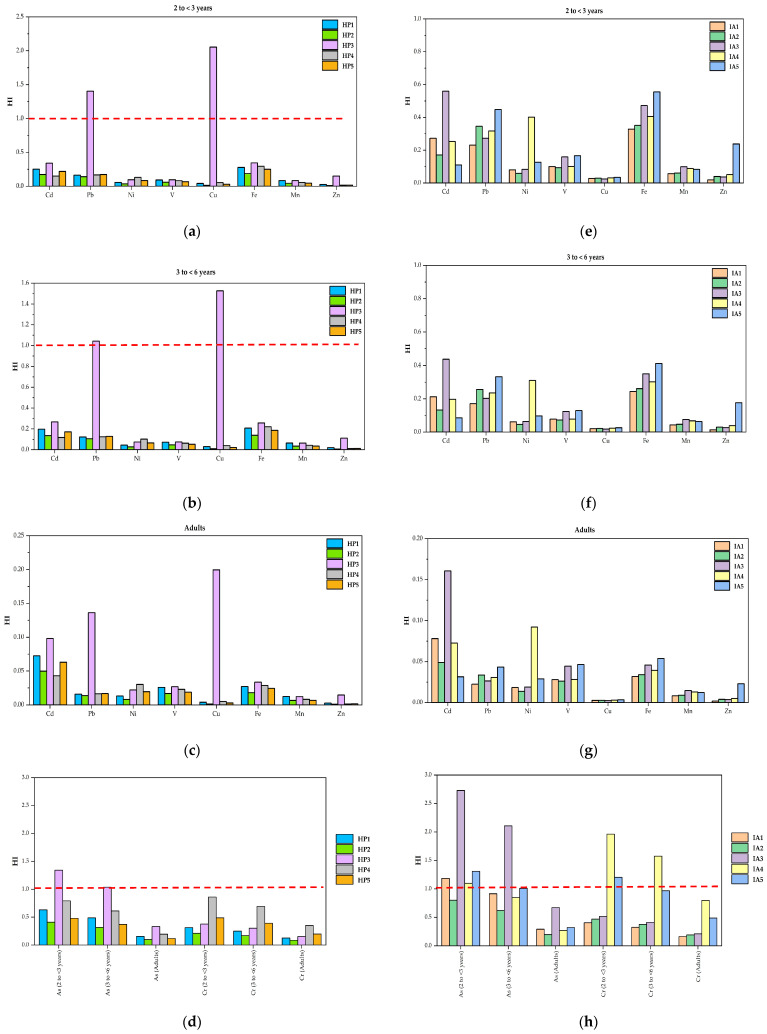
The HI of trace elements in dust inside child development centers in the haze (HP) and industrial areas (IA); (**a**,**e**) children aged 2 to <3 years, (**b**,**f**) children aged 3 to <6 years, (**c**,**g**) adults, and (**d**,**h**) HI values for As and Cr.

Non-carcinogenic risks relate to all adverse health effects in an organism caused by environmental exposures, excluding cancer. The hazard quotient (HQ) was used to assess non-carcinogenic risk in the study of exposure routes to trace elements from dust in indoor child development centers in haze (HP) and industrial areas (IA), as shown in Tables S8–S13. Figure 4a–h illustrates the Hazard Index (HI) values for trace elements in indoor dust exposure at child development centers. The Hazard Quotient (HQ) for all exposure routes to each trace element was below 1.0, indicating no significant no carcinogenic risk in both sampling areas. However, HQ and HI values for arsenic (As), lead (Pb), and copper (Cu) in dust exposure for children aged 2 to <3 years and 3 to <6 years at site HP3 (haze area) exceeded 1.0, indicating the potential for non-carcinogenic health effects. In the industrial area, HI values for arsenic (As) and chromium (Cr) also exceeded 1.0 for the same age groups at sites IA1, IA3, IA4, and IA5, suggesting a possible risk of non-cancer-related health issues, as shown in Figure 4h.

Moreover, the HQ values of trace elements in dust were found to be such that the ingestion exposure route for children’s groups (2 to <3 years and 3 to <6 years) was higher than 1, implying a potential for non-carcinogenic effects to occur. Similar to what was reported by Dubey et al. [14], they found that the highest value of HQ for the ingestion exposure route in children was inside the classroom. In an e-waste recycling area, the HQ for children’s exposure to trace elements in indoor kindergarten dust showed the highest HQ values for the ingestion route of children [12].

However, the majority of HI values resulting from exposure to trace elements in indoor dust exceeded 1, indicating a possible health risk. We found that children in both sampling areas had higher non-carcinogenic risks of trace elements in dust than adults. Xu et al. [12] reported that HI values for children’s exposures to trace elements in dust inside a kindergarten located near an e-waste recycling area were 1.59 for 2–3 years, 1.18 for 3–6 years, and 0.69 for 6–8 years. Indoor kindergartens in Beijing, China, were found to have HI values of trace elements in dust 63 µm for children (0.21) greater than those for adults (7.25 × 10^−3^) [44].

#### 3.4.2. Carcinogenic Risk of Indoor Dust

The cancer risk (CR) for inhalation, ingestion, and dermal exposure routes and the total cancer risk (TCR) were calculated to assess the risk of toxic metals affecting human cancer. Table 6 and Table 7 show the CR and TCR of harmful metals attached to indoor dust that children aged 2 to <3 years, children aged 3 to <6 years, and adults are exposed to at child development centers within the haze (HP) and in industrial areas (IA). Based on CR exposure through eating and skin contact, the study examined the possibility that toxic metals in dust might adversely affect human health in three age groups in both areas. The results indicated that the risk was between 10^−6^ and 10^−4^, indicating that the risk from these metals was considered acceptable or controllable. There is no risk (<10^−6^), even if the inhalation exposure route is suggested. The TCR values for As, Cr, and Ni in indoor dust for three age groups in both areas were between 10^−6^ and 10^−4^, suggesting that the associated cancer risk from these metals was considered an acceptable lifetime. Moreover, Ni had the highest TCR values of trace elements in dust at child development centers in both areas, and it was found more at the IA site than at the HP site. The source of Ni in dust might be emissions from heavy fuel oil combustion industries and exhaust emissions from traffic [59,65]. Moghtaderi et al. [10] revealed that the TCR values of As, Pb, and Cr in school dust from industrial cities in Iran were acceptable carcinogen health risks (10^−6^ and 10^−4^). The estimation of the incremental lifetime carcinogenic risk (ILCR) of As, Cr, and Ni in indoor dust from school and residential areas in China was lower than 10^−6^, indicating a safe carcinogenic risk [11]. Therefore, the carcinogenic risk to children and teachers at child development centers may be concerned with exposure to As, Cr, and Ni in indoor dust.

## 4. Conclusions

This study investigated the levels and health risks of trace elements in indoor dust sampled from child development centers located in haze areas (HP) and industrial areas (IA) in Thailand. Significantly elevated concentrations of hazardous metals, including As, Cr, Pb, V, Fe, Mn, and Zn, were observed between the two environments. The enrichment factor (EF) and the geo-accumulation index (*I_geo_*) results confirmed anthropogenic contributions, especially from vehicular emissions, industrial processes, soil dust, and biomass burning. The potential ecological risk factor ($E_{r}^{i}$) indicated significantly elevated risks, particularly from Cd and Zn in both areas. Principal component analysis (PCA) identified traffic emissions, road dust, and biomass burning as primary sources of pollution in haze areas, whereas traffic and combustion activities were predominant in industrial areas. Non-carcinogenic risk assessments of As, Pb, Cu, and Cr indicated potential health risks (HI > 1) for children in both areas. The total cancer risk (TCR) associated with As, Cr, and Ni is considered tolerable within thresholds of 10^−6^ to 10^−4^; nevertheless, long-term exposure can still lead to chronic health problems. This study emphasizes the need for consistent monitoring and improved regulations concerning the environment in preschool settings. Mitigation strategies, such as improving indoor air quality, reducing dust resuspension, and establishing buffer zones around pollution sources, must be considered. Future research should examine the seasonal and long-term variations in indoor dust composition and its cumulative health impacts.

### Limitations of This Study

1. The investigation was conducted with a limited number of child development centers (5 sampling sites per area) and a relatively small overall number of samples. However, the sample of this research was considered adequate for the investigation undertaken; it may restrict the statistical analysis necessary to relate the source of indoor dust in child development centers. Future research with a high number of samples would enhance the robustness and confidence in observed associations.

2. The sample collection was conducted during a haze period, which did not cover the entire year. This limitation may have excluded seasonal variations, potentially failing to capture the full spectrum of variability in trace element concentrations in indoor dust at child development centers. Such concentrations can fluctuate significantly across different seasons due to changes in weather patterns and episodic pollution events. This constraint is especially relevant given the well-documented annual fluctuations in biomass burning and industrial emissions, both of which can influence trace element levels.

3. The health risk assessment of this study was evaluated from US-EPA data and citations of previous research. Specifically, both non-carcinogenic and carcinogenic risk assessments for children in the child development center should be recorded and calculated based on data collected from the designated study areas.

## Figures and Tables

**Table 1:** Sampling sites and land-use patterns of child development centers.

Site	Code	Classrooms Characteristics	GPS Position Lat-Long
**Haze area (HP)**	HP1	The child development center is located within the community. The distance between the site and the highway is approximately 1.1 km. The building is a single floor with two classrooms. Its building is constructed from concrete materials.	18°15′41.44″ N 99°31′1.405″ E
	HP2	The sampling site is close to a temple area, a nearby alley 8 m wide, and a T-junction road with a high level of traffic intensity. Its buildings consist of a single floor with two classrooms and are constructed from concrete materials.	18°12′29.65″ N 99°29′26.74″ E
	HP3	The child development center is located in front of a primary school area. Buildings consist of a single floor and a classroom. It is constructed from concrete materials. It is near the construction and landfilling supply store. It is approximately 153 m away from a highway.	18°15′25.95″ N 99°28′32.98″ E
	HP4	The location is close to the municipal and government offices. The distance is 362 m from the highway. The structure comprises one level and a classroom. The building is constructed of concrete materials and is located near parking areas.	18°15′48.50″ N 99°28′28.54″ E
	HP5	The sampling site is surrounded by an 8-m-wide alley and T-junction roads. This site is located within a community. Its buildings consist of one level and a classroom and are constructed from concrete materials.	18°16′11.23″ N 99°27′59.45″ E
**Industrial area (IA)**	IA1	The child development center is located within a temple and a primary school. It is surrounded by the community. The structures include a single level and one educational classroom. It is composed of concrete materials and is situated between oil refineries and a coal power plant.	12°40′25.95″ N 101°10′22.08″ E
	IA2	The sampling site is located inside a temple. It is close to the intersection and traffic density during rush hours. It is near petroleum and liquefied natural gas (LNG) plants. Constructed from concrete materials, it spans just one floor and contains a classroom.	12°41′6.575″ N 101°7′0.4794″ E
	IA3	The child development center is located at the primary school of the Maptaphut city municipality. It is built from concrete materials and consists of a single floor with two classrooms. The Eastern Economic Corridor (EEC) project surrounds this site with construction and land leveling for the industrial area.	12°43′59.73″ N 101°7′21.59″ E
	IA4	This site is close to an intersection and a temple. The building comprises a floor and four classrooms, built from concrete materials. It is located close to a tapioca flour factory and local market.	12°45′13.86″ N 101°8′21.14″ E
	IA5	The sampling is in a community near a highway. There is a single floor and a classroom. Its buildings are constructed from concrete materials. It is located close to the steel and iron recycling and natural gas power plants.	12°46′33.61″ N 101°10′44.26″ E

**Table 2:** Concentrations of trace elements in indoor dust (*n* = 3) from the child development centers in haze and industrial areas in Thailand.

Code		Concentrations of Trace Elements in Indoor Dust (mg kg^−1^, *n* = 3)									
		As	Cd	Cr	Pb	Ni	V	Cu	Fe	Mn	Zn
HP1 ^a^	Median	11.1	1.5	43.5	41.8	36.4	27.1	117.1	13,883	637.2	569.6
	Mean **±** SD	11.4 ± 0.5	1.4 ± 0.1	44.7 ± 2.3	41.0 ± 2.5	36.1 ± 0.7	26.7 ± 1.0	115.7 ± 3.5	13,956 ± 378	637.5 ± 6.1	576.1 ± 28.7
HP2 ^a^	Median	7.8	1.0	29.8	35.6	22.9	17.4	37.9	9397	332.3	197.6
	Mean **±** SD	7.4 ± 0.9	1.0 ± 0.1	30.1 ± 1.3	35.4 ± 0.5	22.4 ± 1.5	17.3 ± 0.6	37.8 ± 3.5	9318 ± 184	336.8 ± 14.2	196.4 ± 6.3
HP3 ^a^	Median	24.1	1.9	55.2	347.3	60.5	27.3	5865.1	17,275	630.7	3187.9
	Mean **±** SD	24.2 ± 0.3	2.0 ± 0.1	53.8 ± 5.9	351.1 ± 18.4	60.8 ± 2.3	27.6 ± 0.5	5875 ± 384	17,290 ± 257	630.9 ± 7.2	3203 ± 182
HP4 ^a^	Median	13.8	0.8	127.4	41.7	84.7	24.7	156.3	15,010	422.2	341.4
	Mean **±** SD	14.3 ± 1.0	0.9 ± 0.1	123.4 ± 14.8	41.9 ± 0.6	83.1 ± 2.9	23.6 ± 2.3	150.4 ± 15.0	14,784 ± 442	420.4 ± 8.9	332.4 ± 16.0
HP5 ^a^	Median	8.8	1.2	127.4	41.8	50.5	19.3	87.0	12,386	348.0	356.8
	Mean **±** SD	8.6 ± 0.3	1.2 ± 0.0	69.9 ± 6.0	43.2 ± 2.8	53.5 ± 8.1	19.3 ± 0.5	85.9 ± 5.7	12,523 ± 592	343.7 ± 10.9	349.0 ± 15.4
**Median**		**11.1**	**1.2**	**55.2**	**41.8**	**50.5**	**24.7**	**117.1**	**13,883**	**422.2**	**356.8**
**Mean ± SD**		**13.2 ± 6.2**	**1.3 ± 0.4**	**64.4 ± 34.0**	**102.5 ± 128.9**	**51.2 ± 21.8**	**22.9 ± 4.3**	**1253 ± 2397**	**13,574 ± 2745**	**473.9 ± 139.1**	**931.3 ± 1184**
IA1 ^A^	Median	21.8	1.6	60.1	57.5	51.8	30.4	80.6	16,690	432.9	410.6
	Mean **±** SD	21.3 ± 1.2	1.6 ± 0.1	58.0 ± 6.7	57.7 ± 1.5	50.6 ± 3.6	28.9 ± 3.1	79.9 ± 1.4	16,437 ± 456	428.5 ± 8.8	410.7 ± 10.5
IA2 ^A^	Median	14.1	1.0	65.0	86.8	39.9	27.3	84.0	17,463	459.0	852.7
	Mean **±** SD	14.4 ± 0.8	1.0 ± 0.1	67.2 ± 4.8	86.4 ± 0.8	37.7 ± 4.9	26.9 ± 0.9	84.3 ± 2.4	17,578 ± 248	462.6 ± 8.4	854.7 ± 26.3
IA3 ^A^	Median	47.9	3.1	74.2	66.3	52.2	46.5	67.4	24,796	761.9	783.1
	Mean **±** SD	49.2 ± 2.4	3.2 ± 0.1	74.0 ± 1.0	68.3 ± 4.5	52.5 ± 1.0	45.9 ± 2.3	69.0 ± 3.9	23,619 ± 316	754.4 ± 13.3	781.5 ± 10.6
IA4 ^A^	Median	19.5	1.5	281.8	58.1	253.4	29.9	90.4	20,325	671.0	1099.1
	Mean **±** SD	19.8 ± 0.6	1.4 ± 0.2	281.4 ± 3.5	79.4 ± 37.5	253.5 ± 0.6	29.1 ± 1.5	88.9 ± 3.6	20,310 ± 142	671.1 ± 2.8	1099 ± 1.5
IA5 ^A^	Median	24.0	0.6	166.2	107.0	78.3	47.2	95.7	27,645	628.2	852.7
	Mean **±** SD	23.6 ± 1.1	0.6 ± 0.2	172.5 ± 11.2	112.0 ± 11.5	79.8 ± 3.2	47.8 ± 1.3	99.2 ± 8.9	27,783 ± 420	628.0 ± 9.9	5114 ± 60.3
**Median**		**21.8**	**1.5**	**74.2**	**73.5**	**53.5**	**30.4**	**84.0**	**20,325**	**628.2**	**852.7**
**Mean ± SD**		**25.7 ± 12.6**	**1.6 ± 0.9**	**130.6 ± 89.2**	**80.8 ± 24.2**	**94.8 ± 83.4**	**35.7 ± 9.6**	**84.3 ± 11.1**	**21,146 ± 4296**	**588.9 ± 129.0**	**1652 ± 1806**
***p*-value** ^1^		**<0.001**	0.713	**0.005**	**0.004**	0.217	**<0.001**	0.050	**<0.001**	**0.010**	**0.004**

^1^ Mann–Whitney U test for individual trace elements during the haze and in the industrial area. The values in bold indicate a significant difference (*p*-value < 0.05). ^a, A^ is not a significant difference in the haze and industrial area in each site for using the Kruskal–Wallis test (*p*-value > 0.05).

**Table 3:** Concentrations of trace elements in indoor dust from schools and kindergartens with several studies.

Code	Concentrations of Trace Elements in Indoor Dust (mg kg^−1^ ± SD)										References
	As	Cd	Cr	Pb	Ni	V	Cu	Fe	Mn	Zn	
Haze area in Lampang, Thailand	13.2 ± 6.2	1.3 ± 0.4	64.4 ± 34.0	102.5 ± 128.9	51.2 ± 21.8	22.9 ± 4.3	1253 ± 2397	13,574 ± 2745	473.9 ± 139.1	931.3 ± 1184	This study
Industrial area in Rayong, Thailand	25.7 ± 12.6	1.6 ± 0.9	130.6 ± 89.2	80.8 ± 24.2	94.8 ± 83.4	35.7 ± 9.6	84.3 ± 11.1	21,146 ± 4296	588.9 ± 129.0	1652 ± 1806	This study
Schools in industrial areas, Iran	2.8 ± 1.7	1.0 ± 2.3	172.8 ± 122.1	258.8 ± 268.2	50.1 ± 22.5	-	40.0 ± 22.4	16,946 ± 8691	288.9 ± 156.1	258.8 ± 210.6	[10]
Kindergarten in an E-waste recycling town, China (in 2019 and 2020)	6.8 and 6.3	1.5 and 1.4	155.8 and 1318	153.7 and 92.3	70.0 and 1050	20.2 and 22.6	161.7 and 234.1	-	421.1 and 414.4	2427 and 1352.9	[13]
Schools in an urban area, Lithuania	4.6–70.0	-	60.8–221.2	20.4–564.2	-	26.2–150.9	51.3–395.4	1569–25,649	-	219.5–16,131	[41]
Schools in an industrial area, Puchuncaví, Chile	39.5–331.9	0.6–15.1	373.4–505.4	41.8–324.5	103.8–164.2	330.5–418.4	276.5–54,081	-	636.2–889.5	389.4–1823	[43]
Schools in an industrial area, Quintero, Chile	43.0–76.4	0.6–2.3	307.6–594.8	12.8–67.6	96.1–179.6	293.5–482.5	143.3–1450.6	-	635.8–965.0	199.3–728.6	[43]
Kindergarten in Beijing, China	-	0.4 ± 0.2	81.3 ± 14.8	38.1 ± 18.8	25.6 ± 6.5	78.4 ± 10.3	41.2 ± 16.9	-	614.3 ± 105.0	239.0 ± 112.4	[44]
Urban schools in Lanzhou, China	14.7 ± 1.9	1.5 ± 0.4	70.3 ± 24.4	104.3 ± 31.3	41.8 ± 6.5	-	75.9 ± 26.7	-	-	321.8 ± 32.6	[47]
Rural schools in Lanzhou, China	14.0 ± 5.1	1.86 ± 1.36	75.0 ± 28.7	115.7 ± 60.5	32.3 ± 7.8	-	77.4 ± 29.3	-	-	385.8 ± 221.5	[47]
Standard of soil	6	67	-	400	436.5	-	2920	-	1710	-	[45]
Standard of soil in an industrial area	27	810	640	750	41,000	1000	-	-	32,000	1000	[46]

**Table 4:** The potential ecological risk factor ($E_{r}^{i}$) and the potential ecological risk index (RI) for trace elements in dust in child development centers in haze (HP) and industrial areas (IA).

Code	The Potential Ecological Risk Factor ($E_{r}^{i}$) of Trace Elements in Indoor Dust										RI
	**As**	**Cd**	Cr	Pb	Ni	V	Cu	Fe	Mn	Zn	
HP1	15.14	1442.00	3.54	11.70	13.37	0.95	41.04	0.48	1.88	24.10	1554.0
HP2	9.83	989.20	2.39	10.11	8.31	0.62	13.41	0.32	0.99	8.22	1043.0
HP3	32.25	1950.00	4.27	100.30	22.52	0.98	2083.00	0.59	1.86	134.00	4330.0
HP4	19.02	857.20	9.80	11.96	30.78	0.84	53.34	0.50	1.24	13.91	998.6
HP5	11.52	1253.00	5.55	12.33	19.81	0.69	30.45	0.43	1.01	14.60	1350.0
IA1	28.44	1554.00	4.60	16.49	18.76	1.03	28.32	0.56	1.26	17.19	1671.0
IA2	19.26	974.30	5.34	24.70	13.95	0.96	29.91	0.60	1.36	35.76	1106.0
IA3	65.56	3192.00	5.87	19.52	19.44	1.63	24.47	0.81	2.22	32.70	3364.0
IA4	26.38	1445.00	22.33	22.69	93.88	1.03	31.54	0.69	1.97	45.97	1691.0
IA5	31.42	627.00	13.69	32.01	29.56	1.70	35.18	0.95	1.85	213.98	987.3

**Table 5:** Rotated component matrix of principal component analysis for trace elements in dust in child development centers in haze (HP) and industrial areas (IA).

Trace Elements	HP		IA		
	PC1	PC2	PC1	PC2	PC3
As	**0.915**	0.336	−0.178	**0.960**	−0.192
Cd	**0.919**	−0.238	−0.580	**0.781**	−0.109
Cr	−0.180	**0.978**	0.294	−0.010	**0.954**
Pb	**0.942**	0.012	**0.809**	−0.066	0.091
Ni	0.198	**0.955**	−0.046	−0.038	**0.996**
V	**0.782**	0.320	0.576	**0.774**	−0.176
Cu	**0.946**	0.012	**0.781**	−0.436	0.344
Fe	**0.808**	0.556	**0.772**	0.619	0.107
Mn	**0.820**	−0.009	0.151	**0.870**	0.445
Zn	**0.969**	0.006	**0.977**	0.089	0.040
**Eigenvalues**	6.569	2.285	3.980	3.498	1.946
**% of Variance**	65.7	22.8	39.8	34.9	19.4
**Cumulative %**	65.7	88.5	39.8	74.7	94.2
**Major sources**	Mixed source of traffic emission, road dust, and biomass burning	Industrial emissions/soil dust	Traffic emissions or road dust	Industrial emission/Fuel combustion	Coal -powerplant/soil dust

Factor loading values of >0.700 are shown in bold.

**Table 6:** Cancer risk (CR) and total cancer risk (TCR) related to trace elements exposure in dust inside child development centers in haze areas (HP).

Code	Route	2 to <3 Years					3 to <6 Years					Adult				
		As	Cd	Cr	Pb	Ni	As	Cd	Cr	Pb	Ni	As	Cd	Cr	Pb	Ni
HP1	CR_Inh_	8.85 × 10^−10^	1.40 × 10^−10^	1.22 × 10^−8^	1.12 × 10^−11^	2.13 × 10^−10^	2.24 × 10^−9^	3.55 × 10^−10^	3.08 × 10^−8^	2.82 × 10^−11^	5.39 × 10^−10^	6.56 × 10^−9^	1.04 × 10^−9^	9.03 × 10^−8^	8.28 × 10^−11^	1.58 × 10^−9^
	CR_Ing_	3.38 × 10^−6^	1.09 × 10^−7^	4.44 × 10^−6^	6.91 × 10^−8^	1.22 × 10^−5^	7.53 × 10^−6^	2.42 × 10^−7^	9.89 × 10^−6^	1.54 × 10^−7^	2.71 × 10^−5^	7.39 × 10^−6^	2.38 × 10^−7^	9.70 × 10^−6^	1.51 × 10^−7^	2.66 × 10^−5^
	CR_Dermal_	6.62 × 10^−7^	1.18 × 10^−8^	1.16 × 10^−6^	4.51 × 10^−10^	1.99 × 10^−6^	1.84 × 10^−6^	3.26 × 10^−8^	3.21 × 10^−6^	1.25 × 10^−9^	5.51 × 10^−6^	1.65 × 10^−5^	2.93 × 10^−7^	2.88 × 10^−5^	1.12 × 10^−8^	4.94 × 10^−5^
	TCR	4.04 × 10^−6^	1.21 × 10^−7^	5.61 × 10^−6^	6.96 × 10^−8^	1.42 × 10^−5^	9.36 × 10^−6^	2.75 × 10^−7^	1.31 × 10^−5^	1.55 × 10^−7^	3.26 × 10^−5^	2.39 × 10^−5^	5.31 × 10^−7^	3.86 × 10^−5^	1.62 × 10^−7^	7.60 × 10^−5^
HP2	CR_Inh_	5.74 × 10^−10^	9.64 × 10^−11^	8.22 × 10^−9^	9.66 × 10^−12^	1.33 × 10^−10^	1.45 × 10^−9^	2.43 × 10^−10^	2.08 × 10^−8^	2.44 × 10^−11^	3.35 × 10^−10^	4.26 × 10^−9^	7.15 × 10^−10^	6.09 × 10^−8^	7.16 × 10^−11^	9.83 × 10^−10^
	CR_Ing_	2.19 × 10^−6^	7.46 × 10^−8^	3.00 × 10^−6^	5.97 × 10^−8^	7.57 × 10^−6^	4.88 × 10^−6^	1.66 × 10^−7^	6.67 × 10^−6^	1.33 × 10^−7^	1.68 × 10^−5^	4.79 × 10^−6^	1.63 × 10^−7^	6.55 × 10^−6^	1.30 × 10^−7^	1.65 × 10^−5^
	CR_Dermal_	4.30 × 10^−7^	8.08 × 10^−9^	7.81 × 10^−7^	3.90 × 10^−10^	1.23 × 10^−6^	1.19 × 10^−6^	2.24 × 10^−8^	2.16 × 10^−6^	1.08 × 10^−9^	3.42 × 10^−6^	1.07 × 10^−5^	2.01 × 10^−7^	1.94 × 10^−5^	9.69 × 10^−9^	3.07 × 10^−5^
	TCR	2.62 × 10^−6^	8.28 × 10^−8^	3.79 × 10^−6^	6.01 × 10^−8^	8.80 × 10^−6^	6.08 × 10^−6^	1.89 × 10^−7^	8.85 × 10^−6^	1.34 × 10^−7^	2.03 × 10^−5^	1.55 × 10^−5^	3.65 × 10^−7^	2.60 × 10^−5^	1.40 × 10^−7^	4.72 × 10^−5^
HP3	CR_Inh_	1.89 × 10^−9^	1.90 × 10^−10^	1.47 × 10^−8^	9.58 × 10^−11^	3.59 × 10^−10^	4.76 × 10^−9^	4.80 × 10^−10^	3.71 × 10^−8^	2.42 × 10^−10^	9.08 × 10^−10^	1.40 × 10^−8^	1.41 × 10^−9^	1.09 × 10^−7^	7.10 × 10^−10^	2.66 × 10^−9^
	CR_Ing_	7.20 × 10^−6^	1.47 × 10^−7^	5.35 × 10^−6^	5.93 × 10^−7^	2.05 × 10^−5^	1.60 × 10^−5^	3.27 × 10^−7^	1.19 × 10^−5^	1.32 × 10^−6^	4.57 × 10^−5^	1.57 × 10^−5^	3.21 × 10^−7^	1.17 × 10^−5^	1.29 × 10^−6^	4.48 × 10^−5^
	CR_Dermal_	1.41× 10^−6^	1.59 × 10^−8^	1.40 × 10^−6^	3.87 × 10^−9^	3.35 × 10^−6^	3.91 × 10^−6^	4.41 × 10^−8^	3.87 × 10^−6^	1.07 × 10^−8^	9.28 × 10^−6^	3.51 × 10^−5^	3.96 × 10^−7^	3.47 × 10^−5^	9.62 × 10^−8^	8.32 × 10^−5^
	TCR	8.62 × 10^−6^	1.63 × 10^−7^	6.76 × 10^−6^	5.96 × 10^−7^	2.39 × 10^−5^	2.00 × 10^−5^	3.72 × 10^−7^	1.58 × 10^−5^	1.33 × 10^−6^	5.50 × 10^−5^	5.08 × 10^−5^	7.19 × 10^−7^	4.65 × 10^−5^	1.39 × 10^−6^	1.28 × 10^−4^
HP4	CR_Inh_	1.11 × 10^−9^	8.35 × 10^−11^	3.37 × 10^−8^	1.14 × 10^−11^	4.91 × 10^−10^	2.81 × 10^−9^	2.11 × 10^−10^	8.51 × 10^−8^	2.89 × 10^−11^	1.24 × 10^−9^	8.24 × 10^−9^	6.19 × 10^−10^	2.50 × 10^−7^	8.47 × 10^−11^	3.64 × 10^−9^
	CR_Ing_	4.25 × 10^−6^	6.47 × 10^−8^	1.23 × 10^−5^	7.07 × 10^−8^	2.81 × 10^−5^	9.45 × 10^−6^	1.44 × 10^−7^	2.73 × 10^−5^	1.57 × 10^−7^	6.24 × 10^−5^	9.28 × 10^−6^	1.41 × 10^−7^	2.68 × 10^−5^	1.54 × 10^−7^	6.13 × 10^−5^
	CR_Dermal_	8.32 × 10^−7^	7.00 × 10^−9^	3.20 × 10^−6^	4.61 × 10^−10^	4.58 × 10^−6^	2.31 × 10^−6^	1.94 × 10^−8^	8.87 × 10^−6^	1.28 × 10^−9^	1.27 × 10^−5^	2.07 × 10^−5^	1.74 × 10^−7^	7.95 × 10^−5^	1.15 × 10^−8^	1.14 × 10^−4^
	TCR	5.08 × 10^−6^	7.18 × 10^−8^	1.55 × 10^−5^	7.11 × 10^−8^	3.26 × 10^−5^	1.18 × 10^−5^	1.64 × 10^−7^	3.63 × 10^−5^	1.59 × 10^−7^	7.51 × 10^−5^	3.00 × 10^−5^	3.16 × 10^−7^	1.07 × 10^−4^	1.66 × 10^−7^	1.75 × 10^−4^
HP5	CR_Inh_	6.74 × 10^−10^	1.22 × 10^−10^	1.91 × 10^−8^	1.18 × 10^−11^	3.16 × 10^−10^	1.70 × 10^−9^	3.08 × 10^−10^	4.82 × 10^−8^	2.97 × 10^−11^	7.99 × 10^−10^	4.99 × 10^−9^	9.05 × 10^−10^	1.41 × 10^−7^	8.73 × 10^−11^	2.34 × 10^−9^
	CR_Ing_	2.57 × 10^−6^	9.46 × 10^−8^	6.96 × 10^−6^	7.28 × 10^−8^	1.81 × 10^−5^	5.73 × 10^−6^	2.10 × 10^−7^	1.55 × 10^−5^	1.62 × 10^−7^	4.02 × 10^−5^	5.62 × 10^−6^	2.07 × 10^−7^	1.52 × 10^−5^	1.59 × 10^−7^	3.94 × 10^−5^
	CR_Dermal_	5.04 × 10^−7^	1.02 × 10^−8^	1.81 × 10^−6^	4.75 × 10^−10^	2.95 × 10^−6^	1.40 × 10^−6^	2.84 × 10^−8^	5.03 × 10^−6^	1.32 × 10^−9^	8.17 × 10^−6^	1.25 × 10^−5^	2.54 × 10^−7^	4.51 × 10^−5^	1.18 × 10^−8^	7.32 × 10^−5^
	TCR	3.08 × 10^−6^	1.05 × 10^−7^	8.79 × 10^−6^	7.33 × 10^−8^	2.10 × 10^−5^	7.13 × 10^−6^	2.39 × 10^−7^	2.06 × 10^−5^	1.63 × 10^−7^	4.83 × 10^−5^	1.82 × 10^−5^	4.62 × 10^−7^	6.04 × 10^−5^	1.71 × 10^−7^	1.13 × 10^−4^

**Table 7:** Cancer risk (CR) and total cancer risk (TCR) related to trace element exposure in dust inside child development centers in industrial areas (IA).

Code	Route	2 to <3 Years					3 to <6 Years					Adult				
		As	Cd	Cr	Pb	Ni	As	Cd	Cr	Pb	Ni	As	Cd	Cr	Pb	Ni
IA1	CR_Inh_	1.66 × 10^−9^	1.51 × 10^−10^	1.58 × 10^−8^	1.57 × 10^−11^	2.99 × 10^−10^	4.20 × 10^−9^	3.83 × 10^−10^	4.00 × 10^−8^	3.98 × 10^−11^	7.56 × 10^−10^	1.23 × 10^−8^	1.12 × 10^−9^	1.17 × 10^−7^	1.17 × 10^−10^	2.22 × 10^−9^
	CR_Ing_	6.35 × 10^−6^	1.17 × 10^−7^	5.77 × 10^−6^	9.74 × 10^−8^	1.71 × 10^−5^	1.41 × 10^−5^	2.61 × 10^−7^	1.28 × 10^−5^	2.17 × 10^−7^	3.80 × 10^−5^	1.39 × 10^−5^	2.56 × 10^−7^	1.26 × 10^−5^	2.13 × 10^−7^	3.73 × 10^−5^
	CR_Dermal_	1.24 × 10^−6^	1.27 × 10^−8^	1.50 × 10^−6^	6.36 × 10^−10^	2.79 × 10^−6^	3.45 × 10^−6^	3.52 × 10^−8^	4.17 × 10^−6^	1.76 × 10^−9^	7.73 × 10^−6^	3.09 × 10^−5^	3.15 × 10^−7^	3.74 × 10^−5^	1.58 × 10^−8^	6.93 × 10^−5^
	TCR	7.60 × 10^−6^	1.30 × 10^−7^	7.29 × 10^−6^	9.81 × 10^−8^	1.99 × 10^−5^	1.76 × 10^−5^	2.97 × 10^−7^	1.71 × 10^−5^	2.19 × 10^−7^	4.58 × 10^−5^	4.48 × 10^−5^	5.73 × 10^−7^	5.01 × 10^−5^	2.29 × 10^−7^	1.07 × 10^−4^
IA2	CR_Inh_	1.13 × 10^−9^	9.49 × 10^−11^	1.83 × 10^−8^	2.36 × 10^−11^	2.23 × 10^−10^	2.84 × 10^−9^	2.40 × 10^−10^	4.63 × 10^−8^	5.96 × 10^−11^	5.62 × 10^−10^	8.35 × 10^−9^	7.04 × 10^−10^	1.36 × 10^−7^	1.75 × 10^−10^	1.65 × 10^−9^
	CR_Ing_	4.30 × 10^−6^	7.35 × 10^−8^	6.69 × 10^−6^	1.46 × 10^−7^	1.27 × 10^−5^	9.57 × 10^−6^	1.64 × 10^−7^	1.49 × 10^−5^	3.25 × 10^−7^	2.83 × 10^−5^	9.40 × 10^−6^	1.61 × 10^−7^	1.46 × 10^−5^	3.19 × 10^−7^	2.78 × 10^−5^
	CR_Dermal_	8.42 × 10^−7^	7.95 × 10^−9^	1.74 × 10^−6^	9.52 × 10^−10^	2.07 × 10^−6^	2.34 × 10^−6^	2.21 × 10^−8^	4.83 × 10^−6^	2.64 × 10^−9^	5.75 × 10^−6^	2.09 × 10^−5^	1.98 × 10^−7^	4.33 × 10^−5^	2.37 × 10^−8^	5.16 × 10^−5^
	TCR	5.14 × 10^−6^	8.15 × 10^−8^	8.45 × 10^−6^	1.47 × 10^−7^	1.48 × 10^−5^	1.19 × 10^−5^	1.86 × 10^−7^	1.98 × 10^−5^	3.27 × 10^−7^	3.40 × 10^−5^	3.03 × 10^−5^	3.59 × 10^−7^	5.81 × 10^−5^	3.43 × 10^−7^	7.93 × 10^−5^
IA3	CR_Inh_	3.83 × 10^−9^	3.11 × 10^−10^	2.02 × 10^−8^	1.86 × 10^−11^	3.10 × 10^−10^	9.68 × 10^−9^	7.86 × 10^−10^	5.10 × 10^−8^	4.71 × 10^−11^	7.84 × 10^−10^	2.84 × 10^−8^	2.31 × 10^−9^	1.50 × 10^−7^	1.38 × 10^−10^	2.30 × 10^−9^
	CR_Ing_	1.46 × 10^−5^	2.41 × 10^−7^	7.36 × 10^−6^	1.15 × 10^−7^	1.77 × 10^−5^	3.26 × 10^−5^	5.36 × 10^−7^	1.64 × 10^−5^	2.57 × 10^−7^	3.94 × 10^−5^	3.20 × 10^−5^	5.26 × 10^−7^	1.61 × 10^−5^	2.52 × 10^−7^	3.87 × 10^−5^
	CR_Dermal_	2.87 × 10^−6^	2.61 × 10^−8^	1.92 × 10^−6^	7.52 × 10^−10^	2.89 × 10^−6^	7.95 × 10^−6^	7.23 × 10^−8^	5.32 × 10^−6^	2.09 × 10^−9^	8.01 × 10^−6^	7.13 × 10^−5^	6.48 × 10^−7^	4.77 × 10^−5^	1.87 × 10^−8^	7.19 × 10^−5^
	TCR	1.75 × 10^−5^	2.67 × 10^−7^	9.29 × 10^−6^	1.16 × 10^−7^	2.06 × 10^−5^	4.06 × 10^−5^	6.09 × 10^−7^	2.17 × 10^−5^	2.59 × 10^−7^	4.74 × 10^−5^	1.03 × 10^−4^	1.18 × 10^−6^	6.39 × 10^−5^	2.71 × 10^−7^	1.11 × 10^−4^
IA4	CR_Inh_	1.54 × 10^−9^	1.41 × 10^−10^	7.68 × 10^−8^	2.17 × 10^−11^	1.50 × 10^−9^	3.90 × 10^−9^	3.56 × 10^−10^	1.94 × 10^−7^	5.47 × 10^−11^	3.79 × 10^−9^	1.14 × 10^−8^	1.04 × 10^−9^	5.69 × 10^−7^	1.61 × 10^−10^	1.11 × 10^−8^
	CR_Ing_	5.89 × 10^−6^	1.09 × 10^−7^	2.80 × 10^−5^	1.34 × 10^−7^	8.56 × 10^−5^	1.31 × 10^−5^	2.43 × 10^−7^	6.23 × 10^−5^	2.98 × 10^−7^	1.90 × 10^−4^	1.29 × 10^−5^	2.38 × 10^−7^	6.11 × 10^−5^	2.93 × 10^−7^	1.87 × 10^−4^
	CR_Dermal_	1.15 × 10^−6^	1.18 × 10^−8^	7.29 × 10^−6^	8.75 × 10^−10^	1.40 × 10^−5^	3.20 × 10^−6^	3.27 × 10^−8^	2.02 × 10^−5^	2.43 × 10^−9^	3.87 × 10^−5^	2.87 × 10^−5^	2.93 × 10^−7^	1.81 × 10^−4^	2.18 × 10^−8^	3.47 × 10^−4^
	TCR	7.05 × 10^−6^	1.21 × 10^−7^	3.54 × 10^−5^	1.35 × 10^−7^	9.95 × 10^−5^	1.63 × 10^−5^	2.76 × 10^−7^	8.27 × 10^−5^	3.01 × 10^−7^	2.29 × 10^−4^	4.16 × 10^−5^	5.32 × 10^−7^	2.43 × 10^−4^	3.15 × 10^−7^	5.34 × 10^−4^
IA5	CR_Inh_	1.84 × 10^−9^	6.11 × 10^−11^	4.71 × 10^−8^	3.06 × 10^−11^	4.72 × 10^−10^	4.64 × 10^−9^	1.54 × 10^−10^	1.19 × 10^−7^	7.72 × 10^−11^	1.19 × 10^−9^	1.36 × 10^−8^	4.53 × 10^−10^	3.49 × 10^−7^	2.27 × 10^−10^	3.50 × 10^−9^
	CR_Ing_	7.02 × 10^−6^	4.73 × 10^−8^	1.72 × 10^−5^	1.89 × 10^−7^	2.69 × 10^−5^	1.56 × 10^−5^	1.05 × 10^−7^	3.82 × 10^−5^	4.21 × 10^−7^	6.00 × 10^−5^	1.53 × 10^−5^	1.03 × 10^−7^	3.75 × 10^−5^	4.13 × 10^−7^	5.88 × 10^−4^
	CR_Dermal_	1.37 × 10^−6^	5.12 × 10^−9^	4.47 × 10^−6^	1.23 × 10^−9^	4.40 × 10^−6^	3.81 × 10^−6^	1.42 × 10^−8^	1.24 × 10^−5^	3.42 × 10^−9^	1.22 × 10^−5^	3.42 × 10^−5^	1.27 × 10^−7^	1.11 × 10^−4^	3.07 × 10^−8^	1.09 × 10^−4^
	TCR	8.39 × 10^−6^	5.25 × 10^−8^	2.17 × 10^−5^	1.90 × 10^−7^	3.13 × 10^−5^	1.94 × 10^−5^	1.20 × 10^−7^	5.07 × 10^−5^	4.24 × 10^−7^	7.21 × 10^−5^	4.95 × 10^−5^	2.31 × 10^−7^	1.49 × 10^−4^	4.44 × 10^−7^	1.68 × 10^−4^

## Data Availability

The data that supports the study’s findings are not publicly available owing to sensitivity concerns but are available from the corresponding author upon reasonable request.

## Supplementary Materials

The following supporting information can be downloaded at: https://www.mdpi.com/article/10.3390/toxics13070547/s1, Table S1: Recoveries of elements obtained by the National Institute for Standard and Technology (NIST, USA) Standard Reference Material 1648a (SRM 1648a) and Standard Reference Material 2583 (SRM 2583); Table S2: The limit of detection (LOD) and limit of quantitation (LOQ) of elements; Table S3: Soil background of the mean value of Thailand; Table S4: The classification levels of the geo-accumulation index; Table S5: The classification levels and relation between $E_{r}^{i}$, RI and degree; Table S6: Parameters of health risk assessment through inhalation pathway for cancer metals; Table S7: The reference dose values (RfD) of different exposure pathways, ABSd, GIABS, and SF of elements; Table S8: HQ for 2 to <3 years exposure to trace elements in dust inside child development centers in haze area (HP); Table S9: HQ for 3 to <6 years exposure to trace elements in dust inside child development centers in haze area (HP); Table S10: HQ for adult exposure to trace elements in dust inside child development centers in haze area (HP); Table S11: HQ for 2 to <3 years exposure to trace elements in dust inside child development centers in an industrial area (IA); Table S12: HQ for 3 to <6 years exposure to trace elements in dust inside child development centers in an industrial area (IA); Table S13: HQ for adult exposure to trace elements in dust inside child development centers in an industrial area (IA) [4,27,28,66,67,68,69,70,71,72,73,74,75].
