# Supplementary material for: Trace Elements in Indoor Dust Exposure from Child Development Centers and Health Risk Assessment in Haze and Industrial Areas, Thailand
Source: Toxics. 2025 Jun 29;13(7):547. doi: 10.3390/toxics13070547 (PMC12299625; doi:10.3390/toxics13070547)
Supplement: Supplementary file 1 [file toxics-13-00547-s001.zip › toxics-3648979-supplementary.pdf]

Trace Elements in Indoor Dust Exposure from Child Development Centers and Health Risk Assessment in Haze and Industrial Areas, Thailand

Susira Bootdee[1], Sopittaporn Sillapapiromsuk[2], and Sawaeng Kawichai[3,*]

[1] Chemical Industrial Process and Environment program, Faculty of Science, Energy and Environment, King Mongkut's University of Technology North Bangkok (Rayong campus), Rayong 21120, Thailand; susira.b@sciee.kmutnb.ac.th (S.B.).

[2] Department of Environmental Science and Technology, Faculty of Science, Lampang Rajabhat University, Lampang, Thailand; sopittaporn@g.lpru.ac.th (S.S.).

[3] Environmental and Occupational Health Sciences and Non-Communicable Diseases Center of Excellence, Chiang Mai University, Chiang Mai 50200, Thailand; sawaeng.kaw@cmu.ac.th (S.K.).

Correspondence: Sawaeng Kawichai, E-mail: sawaeng.kaw@cmu.ac.th

Table S1. Recoveries of elements obtained by the National Institute for Standard and Technology (NIST, USA) Standard Reference Material 1648a (SRM 1648a) and Standard Reference Material 2583 (SRM 2583).

Elements	Concentrations (n = 5)			References
	Reference value*	Measured values	% Recovery±SD	
As	7.0 mg kg^{-1}	5.24±0.47 mg kg^{-1}	74.9±6.7	**SRM 2583 (Trace elements in indoor dust)**
Cd	7.3 mg kg^{-1}	6.45±0.95 mg kg^{-1}	88.4±13.1	
Cr	80 mg kg^{-1}	55.91±3.22 mg kg^{-1}	69.9±4.0	
Pb	85.9 mg kg^{-1}	73.58±7.82 mg kg^{-1}	85.7±9.1	
Ni	81.1 mg kg^{-1}	70.72±4.71 mg kg^{-1}	87.2±5.8	**SRM 1648a (Urban particulate matter)**
V	127 mg kg^{-1}	103.5±6.27 mg kg^{-1}	81.5 ±4.9	
Cu	610 mg kg^{-1}	579.2±31.2 mg kg^{-1}	94.9±5.1	
Fe	3.92%	2.96±0.16%	75.6±4.0	
Mn	790 mg kg^{-1}	692.5±3.62 mg kg^{-1}	87.7±4.6	
Zn	4,800 mg kg^{-1}	3,805±190.4 mg kg^{-1}	79.3±4.0	

* Reference concentrations in SRM 2583 (Trace elements in indoor dust) and SRM 1648a (Urban particulate matter)

Table S2. The limit of detection (LOD) and limit of quantitation (LOQ) of elements.

Elements	Concentration (mg kg^{-1})	
	LOD (n = 7)	LOQ (n = 7)
As	0.113	0.376
Cd	0.104	0.346
Cr	0.083	0.277
Pb	0.074	0.248
Ni	0.103	0.344
V	0.067	0.222
Cu	0.051	0.172
Fe	0.178	0.587
Mn	0.087	0.290
Zn	0.200	0.667

Table S3. Soil background of mean value of Thailand.

Elements	Background concentration (mg kg^{-1}) [27]
As	7.50
Cd	0.03
Cr	25.2
Pb	17.5
Ni	13.5
V	56.20[28]
Cu	14.1
Fe	29,300[28]
Mn	340.0[28]
Zn	23.9

Table S4. The classification levels of the geo-accumulation index.

Pollution index	Evaluation criteria	Quality details
EF	EF < 2	Deficiency to minimal enrichment
	2 ≤ EF < 5	Moderate enrichment
	5 ≤ EF < 20	Significant enrichment
	20 ≤ EF < 40	Very high enrichment
	EF ≥ 40	Extremely high enrichment
Igeo	*Igeo*≤ 0	Practically uncontaminated
	0 < *Igeo* ≤ 1	Uncontaminated to moderately contaminated
	1< *Igeo* ≤ 2	Moderately contaminated
	2 < *Igeo* ≤ 3	Moderately to heavily contaminated
	3 < *Igeo* ≤ 4	Heavily contaminated
	4 < *Igeo* ≤ 5	Heavily to extremely contaminated
	Igeo >5	Extremely contaminated

Table S5. The classification levels and relation between E_r^i , RI and degree.

E_r^i	**degree**	**RI**	**degree**
E_r^i <40	low risk	RI<150	low risk
40≤ E_r^i <80	moderate risk	150≤ RI<300	moderate risk
80≤ E_r^i <160	considerable risk	300≤ RI<600	considerable risk
160≤ E_r^i <320	high risk	RI ≥600	high risk
E_r^i ≥320	very high risk		

The reference dose of inhalation ($RfD_{Inhalation}$)

The reference concentrations (RfC) of inhalation are based on the Regional Screening levels (RSLs) Summary Table of United States Environmental Protection Agency (US-EPA) [69], as shown in Table 3. The reference dose of inhalation ($RfD_{Inhalation}$) of indoor dust exposure can be calculated to the RfC and followed as Equation S1 [70].

$$RfD_{Inhalation} = \frac{RfC ? 20 \ m^3 \ day^{-1}}{70 \ Kg} \qquad (S1)$$

The reference dose of dermal (RfD_{Dermal})

The dermal reference dose (RfD_{dermal}) is the dose absorbed with the dermis, which is estimated from the ingestion reference dose ($RfD_{ingestion}$) multiplied by the fraction of contaminants absorbed in the gastrointestinal tract (GIABS) using the specified Equation S2 [71].

$$RfD_{Dermal} = RfD_{Ingestion} ? \ GIABS \qquad (S2)$$

Table S6. The reference dose values (RfD) of different exposure pathways, ABS_d, GIABS and SF of elements.

**Carcinogenic metals**	**$RfD_{Ingestion}$[70] (mg kg^{-1} day^{-1})**	**RfD_{Dermal} (mg kg^{-1} day^{-1})**	**$RfD_{Inhalation}$ (mg kg^{-1} day^{-1})**	**RfC[70] (mg m^{-3})**	**$SF_{Ingestion}$ (per mg kg^{-1} day^{-1})**	**SF_{Dermal} (per mg kg^{-1} day^{-1})**	**$SF_{Inhalation}$ (per mg kg^{-1} day^{-1})**	**ABS_d[70]**	**GIABS[70]**
**As**	3.00E-04	3.00E-04	4.29E-06	1.50E-05	1.50 E+00[69,10]	1.50 E+00[69]	1.20 E+01[72]	0.03	1
**Cd**	1.00E-04	2.50E-06	2.86E-06	1.00 E-05	0.38[74]	6.3[74]	1.50 E+01[72,11]	0.001	0.025
**Cr**	3.00 E-03[73]	3.90E-05	2.86E-05	1.0 E-04	0.501[73]	20[73]	42.0[73]		0.013
**Pb**	3.50E-03[73]	3.50E-03	1.00E-03	0.0035[75]	8.50 E-03 [72,73]	8.50 E-03 [73]	4.20 E-02[72,73]		1
**Ni**	1.10E-02	4.40E-04	4.00E-06	1.40E-05	1.7[73]	42.5[73]	9.10 E-01[72]		0.04
V	5.04 E-03	1.31E-04	2.86E-05	1.00E-04					0.026
Cu	4.00 E-02	4.00E-02	1.14E-02	0.04[75]					1
Fe	7.00 E-01	7.00E-01	2.00E-01	0.7[75]					1
Mn	1.40E-01	1.40E-01	1.43E-05	5.00E-05					1
Zn	3.00E-01	3.00E-01	8.57E-02	0.3[75]					1

Note; The fraction of contaminants absorbed in the gastrointestinal tract (GIABS).

Dermal Absorption Fraction Factors (ABS_d)

Cancer slope factor (SF)

Table S7. Parameters of health risk assessment through inhalation pathway for cancer metals.

Parameters	Meaning and unit	Age category			
		Children		Adults	References
		2 to <3 years	3 to <6 years		
EF	Exposure frequency (days $year^{-1}$)	350	350	350	[66]
ED	Exposure duration (year)	1	3	24	[66,37]
InhR	Inhalation rate (m^3 day^{-1})	8.9	10.1	15.1	[37]
IngR	Ingestion rate (mg day^{-1})	200	200	100	[37]
BW	Bodyweight (kg)	13.8	18.6	75.8	[37]
AT	Averaging time (day)				[4]
	- AT = ED ×365 days for non-carcinogenic risk	365	1,095	8,760	
	-AT = 70 years × 365 days $year^{-1}$ for carcinogenic risk.	25,550	25,550	25,550	
PEF	Particle emission factor (PEF; m^3 kg^{-1})	1.36E+09	1.36E+09	1.36E+09	[66]
SL	Skin adherence factor (mg $(cm^2)^{-1}$)	0.214 (Daycare)	0.214 (Daycare)	0.379 (Soil)	[37]
SA	Exposure skin area (cm^2)	6100	7600	19600	[37]
ABS	Dimensionless dermal absorption factor	0.001 0.03 for As	0.001 0.03 for As	0.001 0.03 for As	[67]
CF	Conversion factor (kg mg^{-1})	10^{-6}	10^{-6}	10^{-6}	[68]

Table S8. HQ for 2 to <3 years exposure to trace elements in dust inside child development centers in haze area (HP).

Code	Route	HQ for 2 to <3 years exposure to trace elements in indoor dust									
		**As**	**Cd**	**Cr**	**Pb**	**Ni**	**V**	**Cu**	**Fe**	**Mn**	**Zn**
HP1	Inh	1.20E-03	2.29E-04	7.10E-04	1.86E-05	4.10E-03	4.25E-04	4.62E-06	3.17E-05	2.03E-02	3.06E-06
	Ing	5.26E-01	2.00E-01	2.07E-01	1.63E-01	4.56E-02	7.36E-02	4.02E-02	2.77E-01	6.33E-02	2.67E-02
	Dermal	1.03E-01	5.23E-02	1.04E-01	1.06E-03	7.44E-03	1.85E-02	2.62E-04	1.81E-03	4.13E-04	1.74E-04
	**HI**	**6.30E-01**	**2.53E-01**	**3.11E-01**	**1.64E-01**	**5.71E-02**	**9.25E-02**	**4.05E-02**	**2.79E-01**	**8.40E-02**	**2.69E-02**
HP2	Inh	7.81E-04	1.57E-04	4.79E-04	1.61E-05	2.55E-03	2.75E-04	1.51E-06	2.12E-05	1.07E-02	1.04E-06
	Ing	3.41E-01	1.37E-01	1.40E-01	1.41E-01	2.83E-02	4.78E-02	1.31E-02	1.85E-01	3.34E-02	9.10E-03
	Dermal	6.68E-02	3.59E-02	7.01E-02	9.17E-04	4.62E-03	1.20E-02	8.57E-05	1.21E-03	2.18E-04	5.94E-05
	**HI**	**4.09E-01**	**1.74E-01**	**2.10E-01**	**1.41E-01**	**3.55E-02**	**6.00E-02**	**1.32E-02**	**1.86E-01**	**4.44E-02**	**9.16E-03**
HP3	Inh	2.56E-03	3.10E-04	8.56E-04	1.60E-04	6.91E-03	4.39E-04	2.34E-04	3.93E-05	2.01E-02	1.70E-05
	Ing	1.12E+00	2.71E-01	2.49E-01	1.39E+00	7.68E-02	7.61E-02	2.04E+00	3.43E-01	6.26E-02	1.48E-01
	Dermal	2.19E-01	7.07E-02	1.25E-01	9.10E-03	1.25E-02	1.91E-02	1.33E-02	2.24E-03	4.09E-04	9.68E-04
	**HI**	**1.34E+00**	**3.42E-01**	**3.75E-01**	**1.40E+00**	**9.62E-02**	**9.56E-02**	**2.05E+00**	**3.46E-01**	**8.31E-02**	**1.49E-01**
HP4	Inh	1.51E-03	1.36E-04	1.96E-03	1.90E-05	9.45E-03	3.75E-04	6.00E-06	3.36E-05	1.34E-02	1.76E-06
	Ing	6.61E-01	1.19E-01	5.72E-01	1.66E-01	1.05E-01	6.50E-02	5.23E-02	2.94E-01	4.17E-02	1.54E-02
	Dermal	1.29E-01	3.11E-02	2.87E-01	1.09E-03	1.71E-02	1.63E-02	3.41E-04	1.92E-03	2.72E-04	1.00E-04
	**HI**	**7.92E-01**	**1.50E-01**	**8.61E-01**	**1.67E-01**	**1.32E-01**	**8.17E-02**	**5.26E-02**	**2.95E-01**	**5.54E-02**	**1.55E-02**
HP5	Inh	9.16E-04	1.99E-04	1.11E-03	1.96E-05	6.08E-03	3.07E-04	3.43E-06	2.85E-05	1.09E-02	1.85E-06
	Ing	4.00E-01	1.74E-01	3.24E-01	1.71E-01	6.76E-02	5.32E-02	2.98E-02	2.49E-01	3.41E-02	1.62E-02
	Dermal	7.84E-02	4.55E-02	1.63E-01	1.12E-03	1.10E-02	1.34E-02	1.95E-04	1.62E-03	2.23E-04	1.06E-04
	**HI**	**4.80E-01**	**2.20E-01**	**4.88E-01**	**1.73E-01**	**8.47E-02**	**6.69E-02**	**3.00E-02**	**2.50E-01**	**4.53E-02**	**1.63E-02**

Note: Inh: Inhalation route, Ing: Ingestion route, and Dermal: Dermal route

Table S9. HQ for 3 to <6 years exposure to trace elements in dust inside child development centers in haze area (HP).

Code	Route	HQ for 3 to <6 years exposure trace elements in indoor dust									
		**As**	**Cd**	**Cr**	**Pb**	**Ni**	**V**	**Cu**	**Fe**	**Mn**	**Zn**
HP1	Inh	1.01E-03	1.93E-04	5.98E-04	1.57E-05	3.45E-03	3.57E-04	3.89E-06	2.67E-05	1.71E-02	2.57E-06
	Ing	3.90E-01	1.49E-01	1.53E-01	1.21E-01	3.38E-02	5.46E-02	2.98E-02	2.06E-01	4.70E-02	1.98E-02
	Dermal	9.52E-02	4.83E-02	9.60E-02	9.81E-04	6.88E-03	1.71E-02	2.43E-04	1.67E-03	3.82E-04	1.61E-04
	**HI**	**4.86E-01**	**1.97E-01**	**2.50E-01**	**1.22E-01**	**4.42E-02**	**7.21E-02**	**3.01E-02**	**2.07E-01**	**6.44E-02**	**2.00E-02**
HP2	Inh	6.58E-04	1.32E-04	4.03E-04	1.36E-05	2.15E-03	2.32E-04	1.27E-06	1.78E-05	9.02E-03	8.78E-07
	Ing	2.53E-01	1.02E-01	1.04E-01	1.04E-01	2.10E-02	3.54E-02	9.75E-03	1.37E-01	2.48E-02	6.75E-03
	Dermal	6.18E-02	3.32E-02	6.48E-02	8.48E-04	4.27E-03	1.11E-02	7.93E-05	1.12E-03	2.02E-04	5.49E-05
	**HI**	**3.16E-01**	**1.35E-01**	**1.69E-01**	**1.05E-01**	**2.74E-02**	**4.67E-02**	**9.83E-03**	**1.38E-01**	**3.40E-02**	**6.81E-03**
HP3	Inh	2.16E-03	2.61E-04	7.21E-04	1.34E-04	5.82E-03	3.69E-04	1.97E-04	3.31E-05	1.69E-02	1.43E-05
	Ing	8.31E-01	2.01E-01	1.85E-01	1.03E+00	5.70E-02	5.65E-02	1.51E+00	2.55E-01	4.65E-02	1.10E-01
	Dermal	2.03E-01	6.54E-02	1.16E-01	8.41E-03	1.16E-02	1.77E-02	1.23E-02	2.07E-03	3.78E-04	8.95E-04
	**HI**	**1.04E+00**	**2.67E-01**	**3.02E-01**	**1.04E+00**	**7.44E-02**	**7.45E-02**	**1.53E+00**	**2.57E-01**	**6.37E-02**	**1.11E-01**
HP4	Inh	1.27E-03	1.15E-04	1.65E-03	1.60E-05	7.96E-03	3.16E-04	5.05E-06	2.83E-05	1.13E-02	1.48E-06
	Ing	4.90E-01	8.84E-02	4.24E-01	1.23E-01	7.79E-02	4.82E-02	3.88E-02	2.18E-01	3.10E-02	1.14E-02
	Dermal	1.20E-01	2.88E-02	2.65E-01	1.00E-03	1.58E-02	1.51E-02	3.15E-04	1.77E-03	2.52E-04	9.29E-05
	**HI**	**6.11E-01**	**1.17E-01**	**6.91E-01**	**1.24E-01**	**1.02E-01**	**6.36E-02**	**3.91E-02**	**2.20E-01**	**4.25E-02**	**1.15E-02**
HP5	Inh	7.71E-04	1.68E-04	9.36E-04	1.65E-05	5.12E-03	2.58E-04	2.88E-06	2.40E-05	9.20E-03	1.56E-06
	Ing	2.97E-01	1.29E-01	2.40E-01	1.27E-01	5.01E-02	3.95E-02	2.21E-02	1.84E-01	2.53E-02	1.20E-02
	Dermal	7.25E-02	4.20E-02	1.50E-01	1.03E-03	1.02E-02	1.23E-02	1.80E-04	1.50E-03	2.06E-04	9.75E-05
	**HI**	**3.70E-01**	**1.71E-01**	**3.92E-01**	**1.28E-01**	**6.54E-02**	**5.21E-02**	**2.23E-02**	**1.86E-01**	**3.47E-02**	**1.21E-02**

Note: Inh: Inhalation route, Ing: Ingestion route, and Dermal: Dermal route

Table S10. HQ for adult exposure to trace elements in dust inside child development centers in haze area (HP).

Code	Route	HQ for adult exposure to trace elements in indoor dust									
		**As**	**Cd**	**Cr**	**Pb**	**Ni**	**V**	**Cu**	**Fe**	**Mn**	**Zn**
HP1	Inh	3.72E-04	7.08E-05	2.19E-04	5.75E-06	1.27E-03	1.31E-04	1.43E-06	9.80E-06	6.26E-03	9.44E-07
	Ing	4.79E-02	1.82E-02	1.88E-02	1.48E-02	4.15E-03	6.70E-03	3.66E-03	2.52E-02	5.76E-03	2.43E-03
	Dermal	1.07E-01	5.42E-02	1.08E-01	1.10E-03	7.71E-03	1.92E-02	2.72E-04	1.87E-03	4.28E-04	1.80E-04
	**HI**	**1.55E-01**	**7.25E-02**	**1.27E-01**	**1.59E-02**	**1.31E-02**	**2.60E-02**	**3.93E-03**	**2.71E-02**	**1.25E-02**	**2.61E-03**
HP2	Inh	2.41E-04	4.86E-05	1.48E-04	4.97E-06	7.87E-04	8.50E-05	4.66E-07	6.54E-06	3.31E-03	3.22E-07
	Ing	3.11E-02	1.25E-02	1.27E-02	1.28E-02	2.58E-03	4.35E-03	1.20E-03	1.68E-02	3.04E-03	8.28E-04
	Dermal	6.93E-02	3.72E-02	7.26E-02	9.50E-04	4.79E-03	1.24E-02	8.88E-05	1.25E-03	2.26E-04	6.15E-05
	**HI**	**1.01E-01**	**4.97E-02**	**8.54E-02**	**1.37E-02**	**8.16E-03**	**1.69E-02**	**1.29E-03**	**1.81E-02**	**6.58E-03**	**8.90E-04**
HP3	Inh	7.92E-04	9.58E-05	2.64E-04	4.93E-05	2.13E-03	1.36E-04	7.24E-05	1.21E-05	6.20E-03	5.25E-06
	Ing	1.02E-01	2.47E-02	2.27E-02	1.27E-01	6.99E-03	6.93E-03	1.86E-01	3.12E-02	5.70E-03	1.35E-02
	Dermal	2.27E-01	7.33E-02	1.30E-01	9.43E-03	1.30E-02	1.98E-02	1.38E-02	2.32E-03	4.23E-04	1.00E-03
	**HI**	**3.30E-01**	**9.81E-02**	**1.53E-01**	**1.36E-01**	**2.21E-02**	**2.69E-02**	**2.00E-01**	**3.36E-02**	**1.23E-02**	**1.45E-02**
HP4	Inh	4.67E-04	4.21E-05	6.06E-04	5.88E-06	2.92E-03	1.16E-04	1.85E-06	1.04E-05	4.13E-03	5.45E-07
	Ing	6.02E-02	1.08E-02	5.21E-02	1.51E-02	9.56E-03	5.92E-03	4.76E-03	2.67E-02	3.80E-03	1.40E-03
	Dermal	1.34E-01	3.22E-02	2.97E-01	1.12E-03	1.78E-02	1.69E-02	3.53E-04	1.98E-03	2.82E-04	1.04E-04
	**HI**	**1.95E-01**	**4.31E-02**	**3.50E-01**	**1.63E-02**	**3.02E-02**	**2.29E-02**	**5.11E-03**	**2.87E-02**	**8.21E-03**	**1.51E-03**
HP5	Inh	2.83E-04	6.16E-05	3.43E-04	6.06E-06	1.88E-03	9.48E-05	1.06E-06	8.79E-06	3.38E-03	5.72E-07
	Ing	3.64E-02	1.59E-02	2.95E-02	1.56E-02	6.15E-03	4.84E-03	2.72E-03	2.26E-02	3.11E-03	1.47E-03
	Dermal	8.12E-02	4.71E-02	1.69E-01	1.16E-03	1.14E-02	1.38E-02	2.02E-04	1.68E-03	2.31E-04	1.09E-04
	**HI**	**1.18E-01**	**6.30E-02**	**1.98E-01**	**1.68E-02**	**1.95E-02**	**1.88E-02**	**2.92E-03**	**2.43E-02**	**6.71E-03**	**1.58E-03**

Note: Inh: Inhalation route, Ing: Ingestion route, and Dermal: Dermal route

Table S11. HQ for 2 to <3 years exposure to trace elements in dust inside child development centers in industrial area (IA).

Code	Route	HQ for 2 to <3 years exposure to trace elements in indoor dust									
		As	Cd	Cr	Pb	Ni	V	Cu	Fe	Mn	Zn
IA1	Inh	2.26E-03	2.47E-04	9.22E-04	2.62E-05	5.76E-03	4.60E-04	3.19E-06	3.74E-05	1.36E-02	2.18E-06
	Ing	9.88E-01	2.16E-01	2.69E-01	2.29E-01	6.40E-02	7.98E-02	2.77E-02	3.26E-01	4.25E-02	1.90E-02
	Dermal	1.94E-01	5.64E-02	1.35E-01	1.50E-03	1.04E-02	2.00E-02	1.81E-04	2.13E-03	2.78E-04	1.24E-04
	**HI**	**1.18E+00**	**2.73E-01**	**4.05E-01**	**2.31E-01**	**8.02E-02**	**1.00E-01**	**2.79E-02**	**3.29E-01**	**5.64E-02**	**1.92E-02**
IA2	Inh	1.53E-03	1.55E-04	1.07E-03	3.93E-05	4.28E-03	4.28E-04	3.36E-06	4.00E-05	1.47E-02	4.54E-06
	Ing	6.69E-01	1.35E-01	3.11E-01	3.43E-01	4.76E-02	7.42E-02	2.93E-02	3.49E-01	4.59E-02	3.96E-02
	Dermal	1.31E-01	3.53E-02	1.56E-01	2.24E-03	7.76E-03	1.86E-02	1.91E-04	2.28E-03	3.00E-04	2.58E-04
	**HI**	**8.02E-01**	**1.71E-01**	**4.69E-01**	**3.46E-01**	**5.96E-02**	**9.33E-02**	**2.95E-02**	**3.51E-01**	**6.09E-02**	**3.99E-02**
IA3	Inh	5.21E-03	5.07E-04	1.18E-03	3.11E-05	5.97E-03	7.29E-04	2.75E-06	5.37E-05	2.40E-02	4.15E-06
	Ing	2.28E+00	4.44E-01	3.43E-01	2.71E-01	6.63E-02	1.27E-01	2.40E-02	4.69E-01	7.49E-02	3.62E-02
	Dermal	4.46E-01	1.16E-01	1.72E-01	1.77E-03	1.08E-02	3.18E-02	1.57E-04	3.06E-03	4.89E-04	2.36E-04
	**HI**	**2.73E+00**	**5.60E-01**	**5.16E-01**	**2.73E-01**	**8.31E-02**	**1.59E-01**	**2.41E-02**	**4.72E-01**	**9.94E-02**	**3.64E-02**
IA4	Inh	2.10E-03	2.30E-04	4.47E-03	3.61E-05	2.88E-02	4.62E-04	3.55E-06	4.62E-05	2.13E-02	5.83E-06
	Ing	9.16E-01	2.01E-01	1.30E+00	3.15E-01	3.20E-01	8.02E-02	3.09E-02	4.03E-01	6.66E-02	5.09E-02
	Dermal	1.79E-01	5.24E-02	6.54E-01	2.06E-03	5.23E-02	2.01E-02	2.02E-04	2.63E-03	4.35E-04	3.32E-04
	**HI**	**1.10E+00**	**2.53E-01**	**1.96E+00**	**3.17E-01**	**4.01E-01**	**1.01E-01**	**3.11E-02**	**4.06E-01**	**8.84E-02**	**5.12E-02**
IA5	Inh	2.50E-03	9.97E-05	2.74E-03	5.09E-05	9.07E-03	7.60E-04	3.96E-06	6.32E-05	2.00E-02	2.71E-05
	Ing	1.09E+00	8.71E-02	7.99E-01	4.45E-01	1.01E-01	1.32E-01	3.45E-02	5.52E-01	6.23E-02	2.37E-01
	Dermal	2.14E-01	2.27E-02	4.01E-01	2.90E-03	1.65E-02	3.31E-02	2.25E-04	3.60E-03	4.07E-04	1.55E-03
	**HI**	**1.31E+00**	**1.10E-01**	**1.20E+00**	**4.48E-01**	**1.26E-01**	**1.66E-01**	**3.47E-02**	**5.55E-01**	**8.27E-02**	**2.38E-01**

Note: Inh: Inhalation route, Ing: Ingestion route, and Dermal: Dermal route

Table S12. HQ for 3 to <6 years exposure to trace elements in dust inside child development centers in industrial area (IA).

Code	Route	HQ for 3 to <6 years exposure to trace elements in indoor dust									
		As	Cd	Cr	Pb	Ni	V	Cu	Fe	Mn	Zn
IA1	Inh	1.90E-03	2.08E-04	7.77E-04	2.21E-05	4.85E-03	3.87E-04	2.68E-06	3.15E-05	1.15E-02	1.83E-06
	Ing	7.33E-01	1.60E-01	1.99E-01	1.70E-01	4.75E-02	5.92E-02	2.06E-02	2.42E-01	3.16E-02	1.41E-02
	Dermal	1.79E-01	5.21E-02	1.25E-01	1.38E-03	9.65E-03	1.85E-02	1.67E-04	1.97E-03	2.57E-04	1.15E-04
	**HI**	**9.14E-01**	**2.13E-01**	**3.25E-01**	**1.71E-01**	**6.20E-02**	**7.81E-02**	**2.08E-02**	**2.44E-01**	**4.33E-02**	**1.42E-02**
IA2	Inh	1.29E-03	1.30E-04	9.00E-04	3.31E-05	3.60E-03	3.60E-04	2.83E-06	3.36E-05	1.24E-02	3.82E-06
	Ing	4.96E-01	1.00E-01	2.31E-01	2.55E-01	3.53E-02	5.51E-02	2.17E-02	2.59E-01	3.41E-02	2.94E-02
	Dermal	1.21E-01	3.27E-02	1.45E-01	2.07E-03	7.18E-03	1.72E-02	1.77E-04	2.11E-03	2.77E-04	2.39E-04
	**HI**	**6.19E-01**	**1.33E-01**	**3.77E-01**	**2.57E-01**	**4.61E-02**	**7.26E-02**	**2.19E-02**	**2.61E-01**	**4.67E-02**	**2.96E-02**
IA3	Inh	4.39E-03	4.27E-04	9.90E-04	2.62E-05	5.02E-03	6.14E-04	2.32E-06	4.52E-05	2.02E-02	3.49E-06
	Ing	1.69E+00	3.29E-01	2.54E-01	2.01E-01	4.92E-02	9.39E-02	1.78E-02	3.48E-01	5.56E-02	2.69E-02
	Dermal	4.12E-01	1.07E-01	1.59E-01	1.64E-03	1.00E-02	2.94E-02	1.45E-04	2.83E-03	4.52E-04	2.18E-04
	**HI**	**2.11E+00**	**4.37E-01**	**4.14E-01**	**2.03E-01**	**6.42E-02**	**1.24E-01**	**1.79E-02**	**3.51E-01**	**7.62E-02**	**2.71E-02**
IA4	Inh	1.77E-03	1.93E-04	3.77E-03	3.04E-05	2.43E-02	3.89E-04	2.99E-06	3.89E-05	1.80E-02	4.91E-06
	Ing	6.80E-01	1.49E-01	9.67E-01	2.34E-01	2.38E-01	5.95E-02	2.29E-02	2.99E-01	4.94E-02	3.78E-02
	Dermal	1.66E-01	4.85E-02	6.05E-01	1.90E-03	4.83E-02	1.86E-02	1.86E-04	2.43E-03	4.02E-04	3.07E-04
	**HI**	**8.48E-01**	**1.98E-01**	**1.58E+00**	**2.36E-01**	**3.10E-01**	**7.85E-02**	**2.31E-02**	**3.02E-01**	**6.78E-02**	**3.81E-02**
IA5	Inh	2.10E-03	8.39E-05	2.31E-03	4.29E-05	7.64E-03	6.40E-04	3.33E-06	5.32E-05	1.68E-02	2.28E-05
	Ing	8.10E-01	6.46E-02	5.93E-01	3.30E-01	7.48E-02	9.78E-02	2.56E-02	4.09E-01	4.62E-02	1.76E-01
	Dermal	1.98E-01	2.10E-02	3.71E-01	2.68E-03	1.52E-02	3.06E-02	2.08E-04	3.33E-03	3.76E-04	1.43E-03
	**HI**	**1.01E+00**	**8.58E-02**	**9.66E-01**	**3.33E-01**	**9.77E-02**	**1.29E-01**	**2.58E-02**	**4.13E-01**	**6.34E-02**	**1.77E-01**

Note: Inh: Inhalation route, Ing: Ingestion route, and Dermal: Dermal route

Table S13. HQ for adult exposure to trace elements in dust inside child development centers in industrial area (IA).

Code	Route	HQ for adult exposure to trace elements in indoor dust									
		As	Cd	Cr	Pb	Ni	V	Cu	Fe	Mn	Zn
IA1	Inh	6.98E-04	7.63E-05	2.85E-04	8.11E-06	1.78E-03	1.42E-04	9.84E-07	1.15E-05	4.21E-03	6.73E-07
	Ing	9.00E-02	1.97E-02	2.45E-02	2.09E-02	5.82E-03	7.26E-03	2.53E-03	2.97E-02	3.87E-03	1.73E-03
	Dermal	2.00E-01	5.84E-02	1.40E-01	1.55E-03	1.08E-02	2.08E-02	1.88E-04	2.21E-03	2.88E-04	1.29E-04
	**HI**	**2.91E-01**	**7.82E-02**	**1.65E-01**	**2.24E-02**	**1.84E-02**	**2.82E-02**	**2.71E-03**	**3.19E-02**	**8.37E-03**	**1.86E-03**
IA2	Inh	4.73E-04	4.78E-05	3.30E-04	1.21E-05	1.32E-03	1.32E-04	1.04E-06	1.23E-05	4.54E-03	1.40E-06
	Ing	6.09E-02	1.23E-02	2.83E-02	3.12E-02	4.33E-03	6.76E-03	2.67E-03	3.18E-02	4.18E-03	3.60E-03
	Dermal	1.36E-01	3.66E-02	1.62E-01	2.32E-03	8.04E-03	1.93E-02	1.98E-04	2.36E-03	3.10E-04	2.68E-04
	**HI**	**1.97E-01**	**4.90E-02**	**1.91E-01**	**3.36E-02**	**1.37E-02**	**2.62E-02**	**2.87E-03**	**3.41E-02**	**9.03E-03**	**3.87E-03**
IA3	Inh	1.61E-03	1.57E-04	3.63E-04	9.59E-06	1.84E-03	2.25E-04	8.50E-07	1.66E-05	7.41E-03	1.28E-06
	Ing	2.07E-01	4.04E-02	3.12E-02	2.47E-02	6.04E-03	1.15E-02	2.18E-03	4.27E-02	6.82E-03	3.30E-03
	Dermal	4.62E-01	1.20E-01	1.78E-01	1.83E-03	1.12E-02	3.29E-02	1.62E-04	3.17E-03	5.06E-04	2.45E-04
	**HI**	**6.71E-01**	**1.61E-01**	**2.10E-01**	**2.65E-02**	**1.91E-02**	**4.47E-02**	**2.35E-03**	**4.59E-02**	**1.47E-02**	**3.54E-03**
IA4	Inh	6.48E-04	7.10E-05	1.38E-03	1.12E-05	8.90E-03	1.43E-04	1.10E-06	1.43E-05	6.59E-03	1.80E-06
	Ing	8.34E-02	1.83E-02	1.19E-01	2.87E-02	2.92E-02	7.30E-03	2.81E-03	3.67E-02	6.06E-03	4.63E-03
	Dermal	1.86E-01	5.43E-02	6.78E-01	2.13E-03	5.41E-02	2.09E-02	2.09E-04	2.73E-03	4.50E-04	3.44E-04
	**HI**	**2.70E-01**	**7.27E-02**	**7.98E-01**	**3.09E-02**	**9.22E-02**	**2.83E-02**	**3.02E-03**	**3.94E-02**	**1.31E-02**	**4.98E-03**
IA5	Inh	7.71E-04	3.08E-05	8.47E-04	1.57E-05	2.80E-03	2.35E-04	1.22E-06	1.95E-05	6.17E-03	8.38E-06
	Ing	9.94E-02	7.93E-03	7.27E-02	4.05E-02	9.18E-03	1.20E-02	3.14E-03	5.02E-02	5.67E-03	2.16E-02
	Dermal	2.21E-01	2.36E-02	4.16E-01	3.01E-03	1.70E-02	3.43E-02	2.33E-04	3.73E-03	4.22E-04	1.60E-03
	**HI**	**3.22E-01**	**3.15E-02**	**4.89E-01**	**4.35E-02**	**2.90E-02**	**4.65E-02**	**3.37E-03**	**5.40E-02**	**1.23E-02**	**2.32E-02**

Note: Inh: Inhalation route, Ing: Ingestion route, and Dermal: Dermal route
